# Child–Pugh Versus MELD Score for the Assessment of Prognosis in Liver Cirrhosis: A Systematic Review and Meta-Analysis of Observational Studies

**DOI:** 10.1097/MD.0000000000002877

**Published:** 2016-03-03

**Authors:** Ying Peng, Xingshun Qi, Xiaozhong Guo

**Affiliations:** From the Liver Cirrhosis Study Group, Department of Gastroenterology, General Hospital of Shenyang Military Area, Shenyang (YP, XQ, XG); and Postgraduate College, Dalian Medical University, Dalian, China (YP).

## Abstract

Child–Pugh and MELD scores have been widely used for the assessment of prognosis in liver cirrhosis. A systematic review and meta-analysis aimed to compare the discriminative ability of Child–Pugh versus MELD score to assess the prognosis of cirrhotic patients.

PubMed and EMBASE databases were searched. The statistical results were summarized from every individual study. The summary areas under receiver operating characteristic curves, sensitivities, specificities, positive and negative likelihood ratios, and diagnostic odds ratios were also calculated.

Of the 1095 papers initially identified, 119 were eligible for the systematic review. Study population was heterogeneous among studies. They included 269 comparisons, of which 44 favored MELD score, 16 favored Child–Pugh score, 99 did not find any significant difference between them, and 110 did not report the statistical significance. Forty-two papers were further included in the meta-analysis. In patients with acute-on-chronic liver failure, Child–Pugh score had a higher sensitivity and a lower specificity than MELD score. In patients admitted to ICU, MELD score had a smaller negative likelihood ratio and a higher sensitivity than Child–Pugh score. In patients undergoing surgery, Child–Pugh score had a higher specificity than MELD score. In other subgroup analyses, Child–Pugh and MELD scores had statistically similar discriminative abilities or could not be compared due to the presence of significant diagnostic threshold effects.

Although Child–Pugh and MELD scores had similar prognostic values in most of cases, their benefits might be heterogeneous in some specific conditions. The indications for Child–Pugh and MELD scores should be further identified.

## Abstract 2

Supplemental Digital Content is available in the text

## INTRODUCTION

Liver cirrhosis has a high morbidity and mortality, which is the 14th most common cause of death all over the world and the 4th in central Europe. It leads to 1.03 million deaths per year in the world,^1^ and 170,000 deaths per year in Europe.^2^ The prevalence of liver cirrhosis may be underestimated, because patients at the early phase of liver cirrhosis are often asymptomatic, and most of patients with liver cirrhosis are admitted due to its related complications. The 1-year mortality of liver cirrhosis varies greatly from 1% to 57% according to the complications.^3^ It is necessary to use the prognostic models to identify high-risk patients.

Child–Pugh score was firstly proposed by Child and Turcotte to predict the operative risk in patients undergoing portosystemic shunt surgery for variceal bleeding. The primary version of Child–Pugh score included ascites, hepatic encephalopathy (HE), nutritional status, total bilirubin, and albumin. Pugh et al^4^ modified the Child–Pugh classification by adding prothrombin time or international normalized ratio (INR) and removing nutritional status. Child–Pugh score has been widely used to assess the severity of liver dysfunction in clinical work.

Model for end-stage liver disease (MELD) score was initially created to predict the survival of patients undergoing transjugular intrahepatic portosystemic shunts (TIPS).^5^ The primary version of MELD score included the etiology of liver cirrhosis, but this variable was unnecessary.^6^ The present version of MELD score incorporated only 3 objective variables, including total bilirubin, creatinine, and INR. Currently, it has been used to rank the priority of liver transplantation (LT) candidates.

Child–Pugh and MELD scores have been widely used to predict the outcomes of cirrhotic patients. However, they have some drawbacks. First, 2 variables (i.e., ascites and HE) included in Child–Pugh score are subjective and may be variable according to the physicians’ judgment and the use of diuretics and lactulose. Second, INR, which is one component of both Child-Pugh and MELD scores, does not sufficiently reflect coagulopathy and consequently liver function in liver cirrhosis.^7^ Third, there is an interlaboratory variation in INR value.^8^

Until now, a large number of studies compared their discriminative abilities. But the results remained controversial. Some studies favored the Child–Pugh score, but the others were on the opposite side. The aim of this systematic review and meta-analysis was to compare the discriminative ability of Child–Pugh versus MELD score for the assessment of prognosis in cirrhotic patients.

## METHODS

This work is registered on PROSPERO database (registration number: CRD42015019700). Because this work is a systematic review of literatures, the ethical approval and patient consent are not necessary.

### Study Search and Selection

We searched the PubMed and EMBASE databases. The search terms were as follows: (“Child score” or “Child–Pugh score” or “Child–Turcotte–Pugh score”) and (“MELD score” or “model for end stage liver disease score”) and (“liver cirrhosis”). The last search was performed on April 20, 2015.

The inclusion criteria were as follows: patients had been definitely diagnosed as liver cirrhosis; both Child–Pugh and MELD scores were calculated; areas under receiver operating characteristic curve of Child–Pugh versus MELD scores were compared; and sensitivity, specificity, and number of patients with endpoint events were reported. We excluded the following papers: duplicated papers; case reports; reviews; letters; commentaries; corrections; and papers unrelated to comparison of Child–Pugh and MELD scores. We did not restrict the publication years or study design.

### Data Extraction

We extracted the following data: First author, study design, regions of study, the number of patients and the number of patients analyzed, age, sex, study population, etiology of cirrhosis, proportion of hepatocellular carcinoma (HCC), endpoints, cut-off value, true positive value, false positive value, false negative value, and true negative value.

### Quality Assessment

Quality Assessment of Diagnostic Accuracy Studies (QUADAS) 2, a revised version of QUADAS, was used for the quality assessment.^9^ We obtained the detailed information of the QUADAS 2 tool from the website (www.quadas.org). There are 4 key aspects incorporated: patient selection, index test, reference standard, and flow and timing. In the former 3 aspects, the risk of bias and applicability should be evaluated. In the last one, only the risk of bias should be evaluated. The risk of bias is judged as “low,” “high,” or “unclear.” If all the answers are “yes,” it should be judged as “low” risk. If any answer is “unclear,” it should be judged as “unclear” risk. If all answers are “no,” it should be judged as “high” risk. Similarly, the applicability is classified as “low concern,” “high concern,” or “unclear concern.” If the relevant information was not given, it would be classified as “unclear concern.”

### Meta-Analysis

The true positive, false positive, false negative, and true negative values were extracted and entered into the Meta-DiSc software version 1.4. If the diagnostic threshold effect was not statistically significant (*P* > 0.05 in the Spearman correlation test), the diagnostic accuracy would be further evaluated by a random-effects model. The summary areas under receiver operating characteristic curves (AUSROCs) with standard errors (SEs) and Q indexes with SEs, summary sensitivities and specificities with 95% confidence intervals (CIs), summary positive and negative likelihood ratios (PLRs and NLRs) with 95%CIs, and summary diagnostic odds ratios (DORs) with 95%CIs were reported. A statistically significant difference between the 2 scores was evaluated by analyzing the lower and upper limits of 95%CIs. If the diagnostic threshold effect was statistically significant (*P* < 0.05 in the Spearman correlation test), only AUSROCs with SEs and Q indexes with SEs were reported, but not sensitivities, specificities, PLRs, NLRs, or DORs. The heterogeneity among studies was evaluated by Chi-square test and inconsistency index. *P* < 0.1 and/or *I*^2^ > 50% was suggestive of considerable heterogeneity.

## RESULTS

### Paper Selection

**FIGURE 1:**
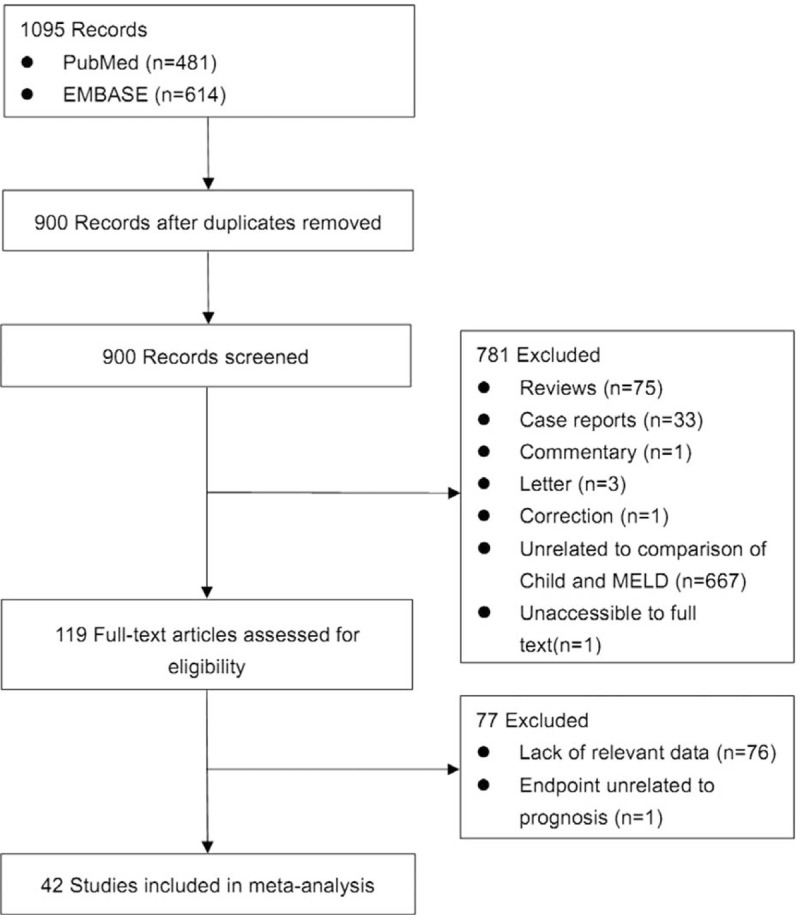
Flowchart of study inclusion.

Overall, 1095 papers were identified via the 2 databases. According to the eligibility criteria, 119 papers were eligible for the systematic review (Figure 1).^10–128^

### Description of Study Characteristics

The characteristics of the 119 papers were shown in Table 1      . The countries included Austria (n = 1),^11^ Belgium (n = 2),^38,96^ China (n = 26),^20,21,27,30,31,53–55,59,60,74,84,102,109,112,113,117,119–121,123–128^ Cuba (n = 1),^47^ Czech Republic (n = 1),^44^ Egypt (n = 1),^51^ France (n = 6),^25,37,41,71,77,114^ Germany (n = 7),^12,48–50,92,105,111^ Greece (n = 1),^82^ Hungary (n = 1),^61^ India (n = 10),^19,29,39,40,67,75,76,86,98,115^ Iran (n = 1),^87^ Italy (n = 5),^22,24,43,46,91^ Ivory Coast (n = 1),^13^ Japan (n = 2),^57,106^ Mexico (n = 1),^45^ Nepal (n = 1),^28^ Pakistan (n = 2),^62,97^ Poland (n = 1),^88^ Portugal (n = 3),^23,26,36^ Serbia (n = 1),^18^ Singapore (n = 2),^72,73^ South Korea (n = 17),^10,15,16,32,33,56,63–66,68–70,83,99,100,103^ Spain (n = 7),^14,58,89,90,94,95,116^ Tunisia (n = 1),^78^ Turkey (n = 3),^80,107,108^ UK (n = 3),^34,42,110^ and USA (n = 11).^17,35,52,79,81,85,93,101,104,118,122^ The total number of patients analyzed in the included studies was 29,414. The number of patients varied from 17 to 2271.

**TABLE 1:**
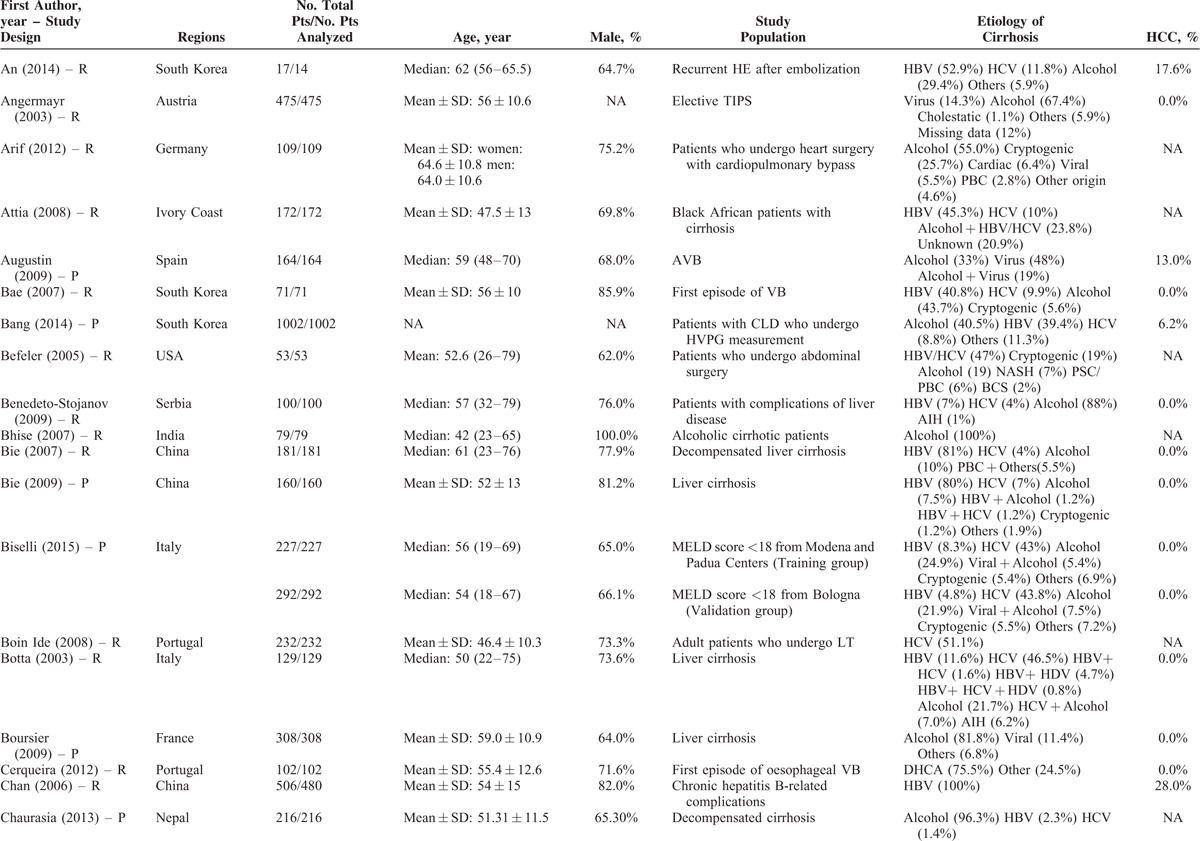
Study Characteristics: An Overview of Studies

**TABLE 1 (Continued):**
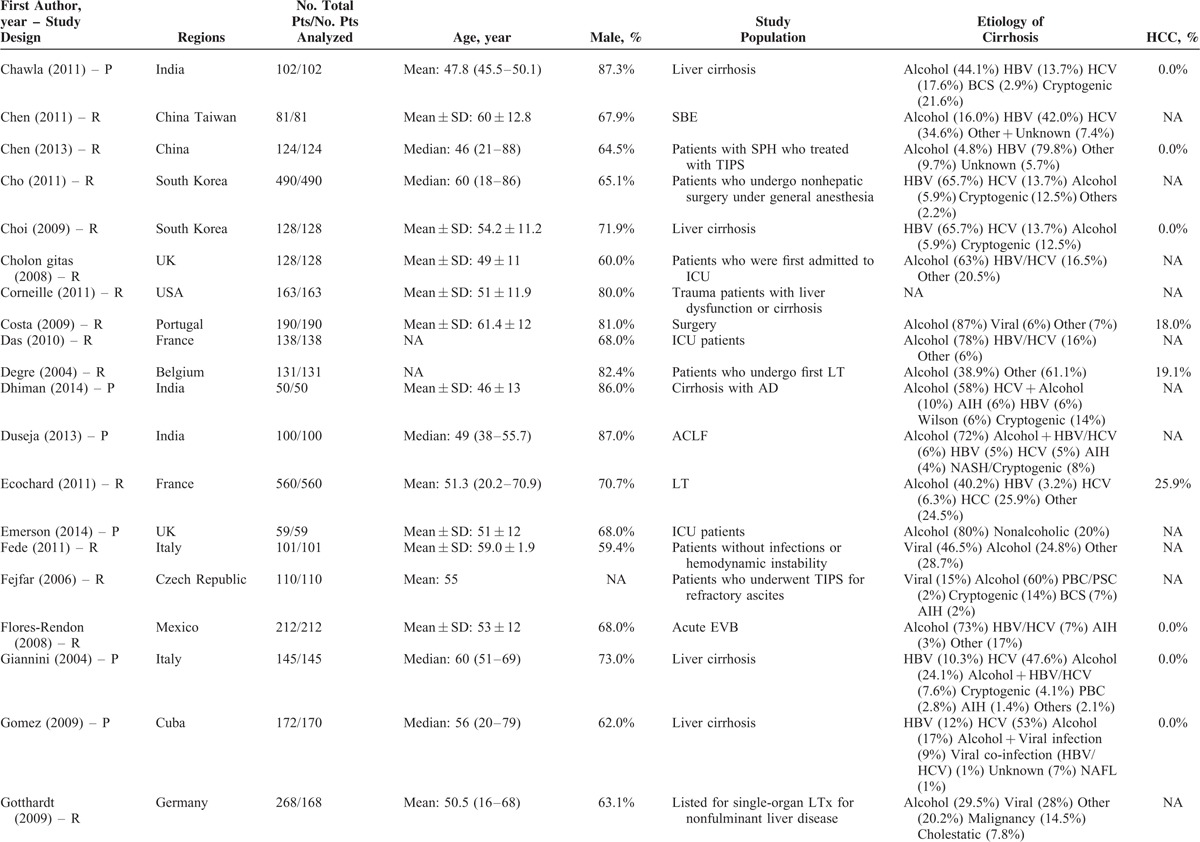
Study Characteristics: An Overview of Studies

**TABLE 1 (Continued):**
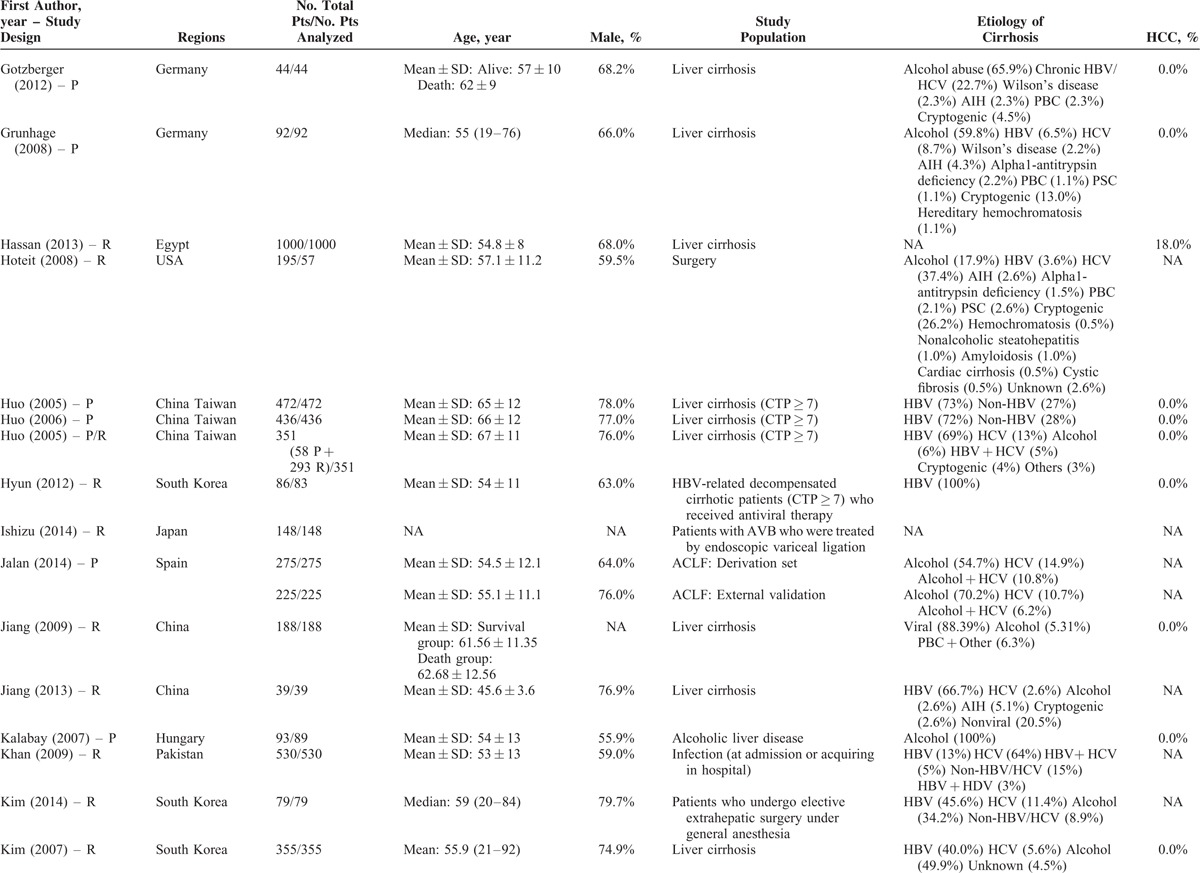
Study Characteristics: An Overview of Studies

**TABLE 1 (Continued):**
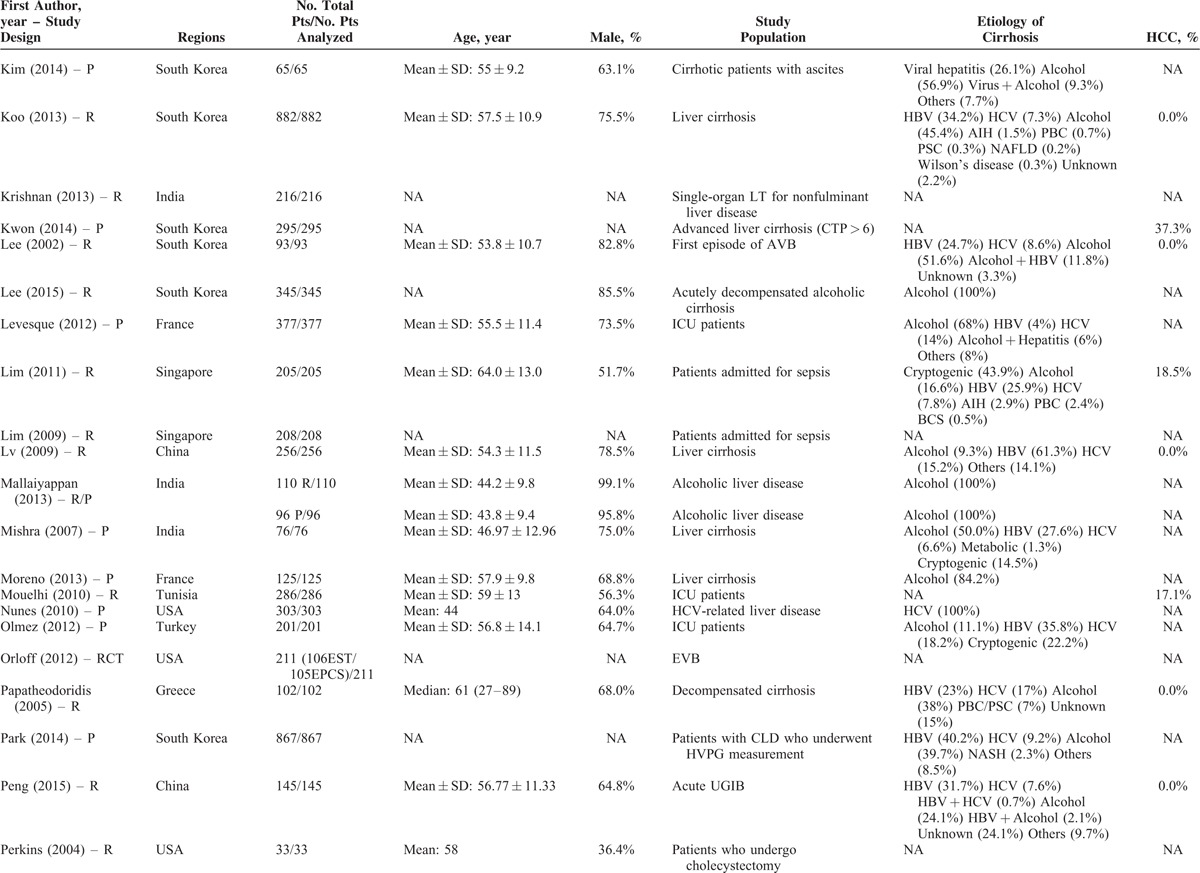
Study Characteristics: An Overview of Studies

**TABLE 1 (Continued):**
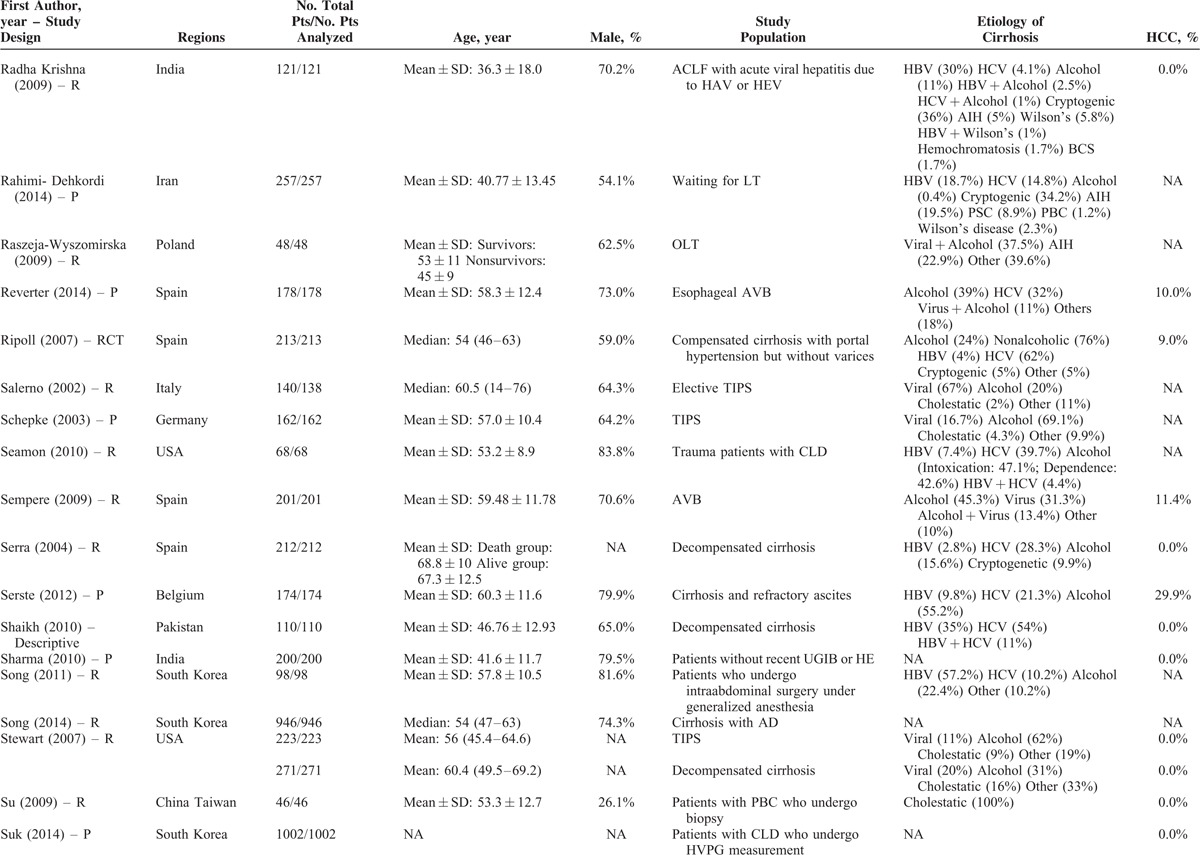
Study Characteristics: An Overview of Studies

**TABLE 1 (Continued):**
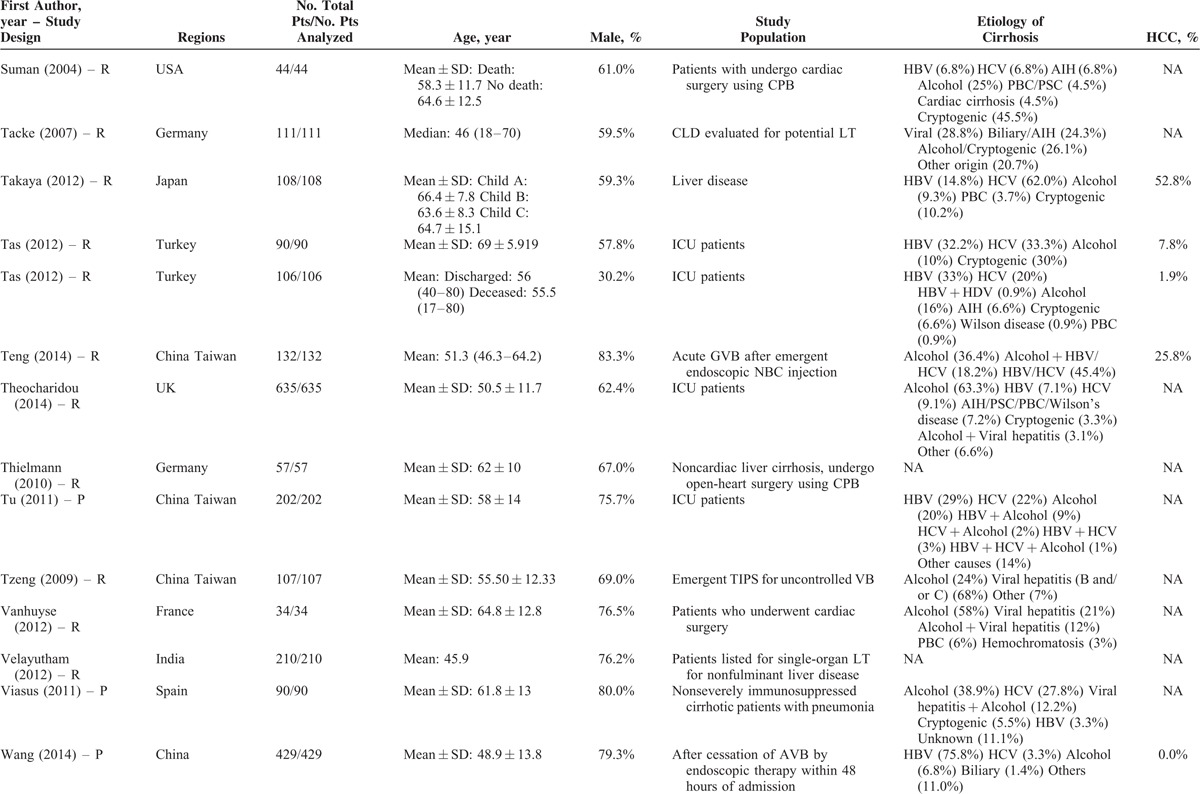
Study Characteristics: An Overview of Studies

**TABLE 1 (Continued):**
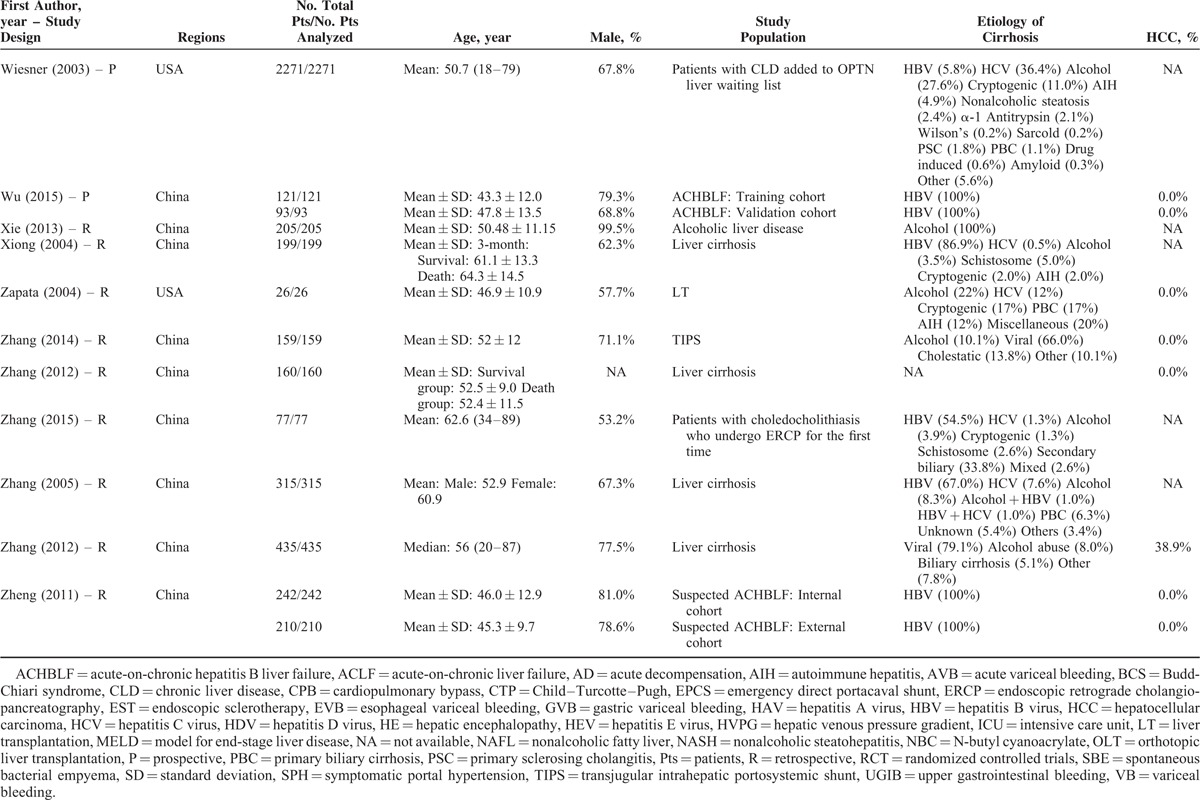
Study Characteristics: An Overview of Studies

The characteristics of study population were heterogeneous among studies. According to the clinical presentations, etiology of liver diseases, patients’ conditions, and treatment options, they were mainly classified as follows: patients presenting with acute gastrointestinal bleeding (n = 12),^14,15,26,45,57,69,81,84,89,94,109,117^ patients presenting with ascites (n = 2),^65,96^ patients presenting with HE (n = 1),^10^ patients presenting with acute-on-chronic liver failure (ACLF) (n = 5),^40,58,86,119,128^ patients presenting with infection, sepsis, or spontaneous bacterial empyema (n = 5),^30,62,72,73,116^ patients admitted to intensive care unit (ICU) (n = 10),^34,37,42,71,78,80,107,108,110,112^ patients with trauma (n = 2),^35,93^ patients with viral hepatitis-related liver cirrhosis alone (n = 3),^27,56,79^ patients with alcohol-related liver cirrhosis alone (n = 5),^19,61,70,75,120^ patients undergoing TIPS (n = 8),^11,31,44,91,92,101,113,123^ patients undergoing LT (n = 10),^23,38,41,48,67,87,88,105,115,122^ patients undergoing abdominal, cardiac, or other surgery/procedure (n = 13),^12,17,32,36,52,63,85,99,102,104,111,114,125^ and unselected patients with liver cirrhosis (n = 43).^13,16,18,20–22,24,25,28,29,33,39,43,46,47,49,51,53–55,59,60,64,66,68,74,76,77,82,83,90,95,97,98,100,103,106,118,121,124,126,127^ In 42 studies, no patient with HCC was included;^11,15,18,20–22,24–26,29,31,33,45–47,49,50,53–56,59,61,64,66,69,74,82,84,86,95,97,98,101–103,117,119,122–124,128^ in 57 studies, the information regarding the number of patients with HCC was lacking;^12,13,17,19,23,28,30,32,34,35,37,39,40,42–44,48,52,57,58,60,62,63,65,67,70,71,73,75–77,79–81,83,85,87,88,91–93,99,100,104,105,110–116,118,120,121,125,126^ and in 20 studies, 1.9% to 52.8% of included patients were diagnosed with HCC.^10,14,16,27,36,38,41,51,68,72,78,89,90,94,96,106–109,127^

### Description of Statistical Results

Their statistical results were summarized in Table 2         . There were 269 comparisons between MELD and Child–Pugh scores. Among 60 comparisons, a statistically significant difference (*P* < 0.05) was observed. In details, the superiority of MELD score over Child–Pugh score was observed in 44 comparisons; and the superiority of Child–Pugh score over MELD score was observed in 16 comparisons. Among 99 comparisons, no statistically significant difference (*P* ≥ 0.05) was observed. Among 110 comparisons, the statistical significance was not reported.

**TABLE 2:**
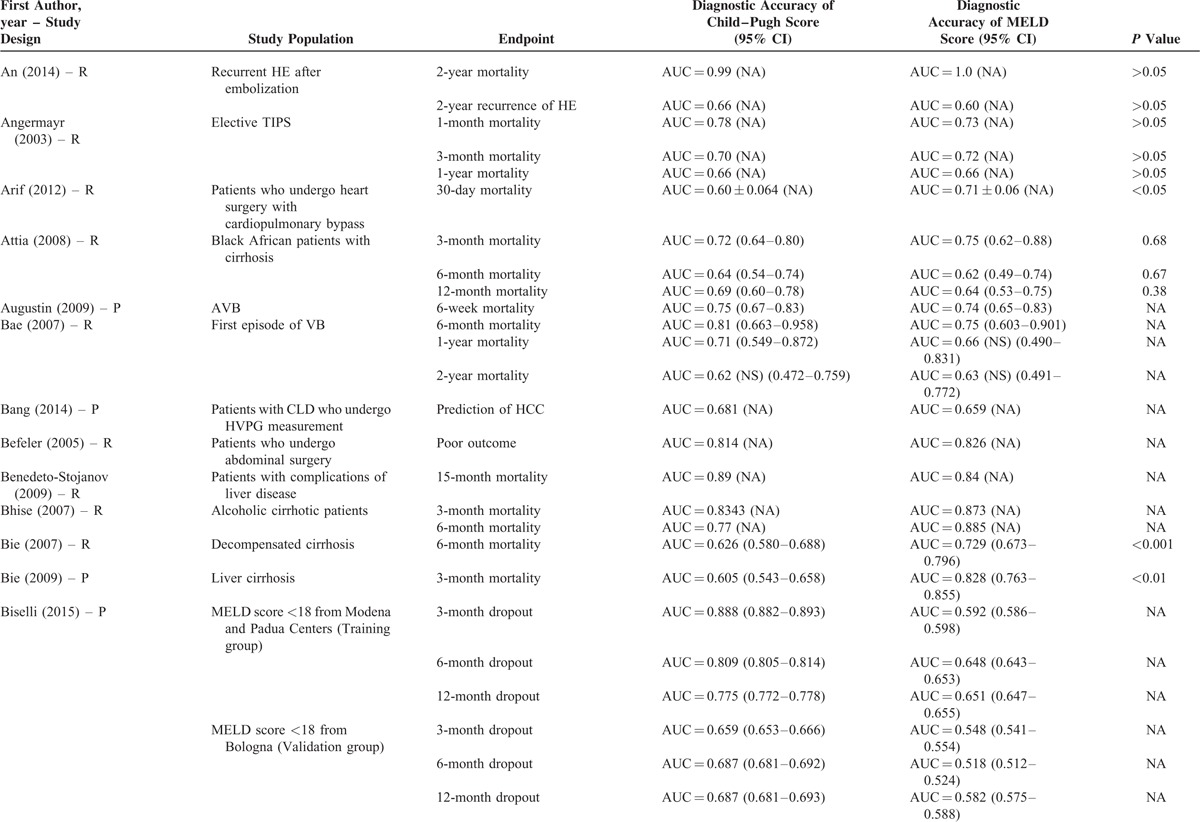
Results of Comparison Between MELD and Child–Pugh Score: An Overview of Studies

**TABLE 2 (Continued):**
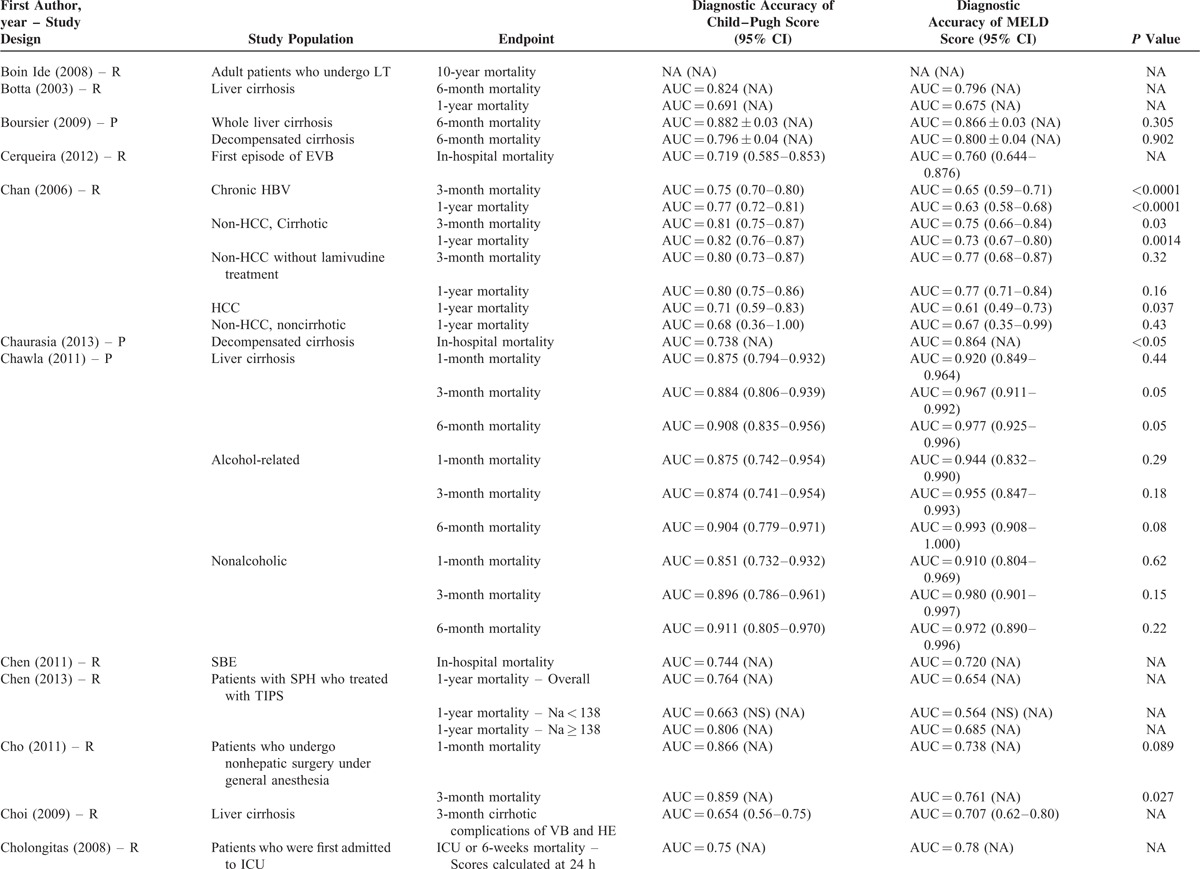
Results of Comparison Between MELD and Child–Pugh Score: An Overview of Studies

**TABLE 2 (Continued):**
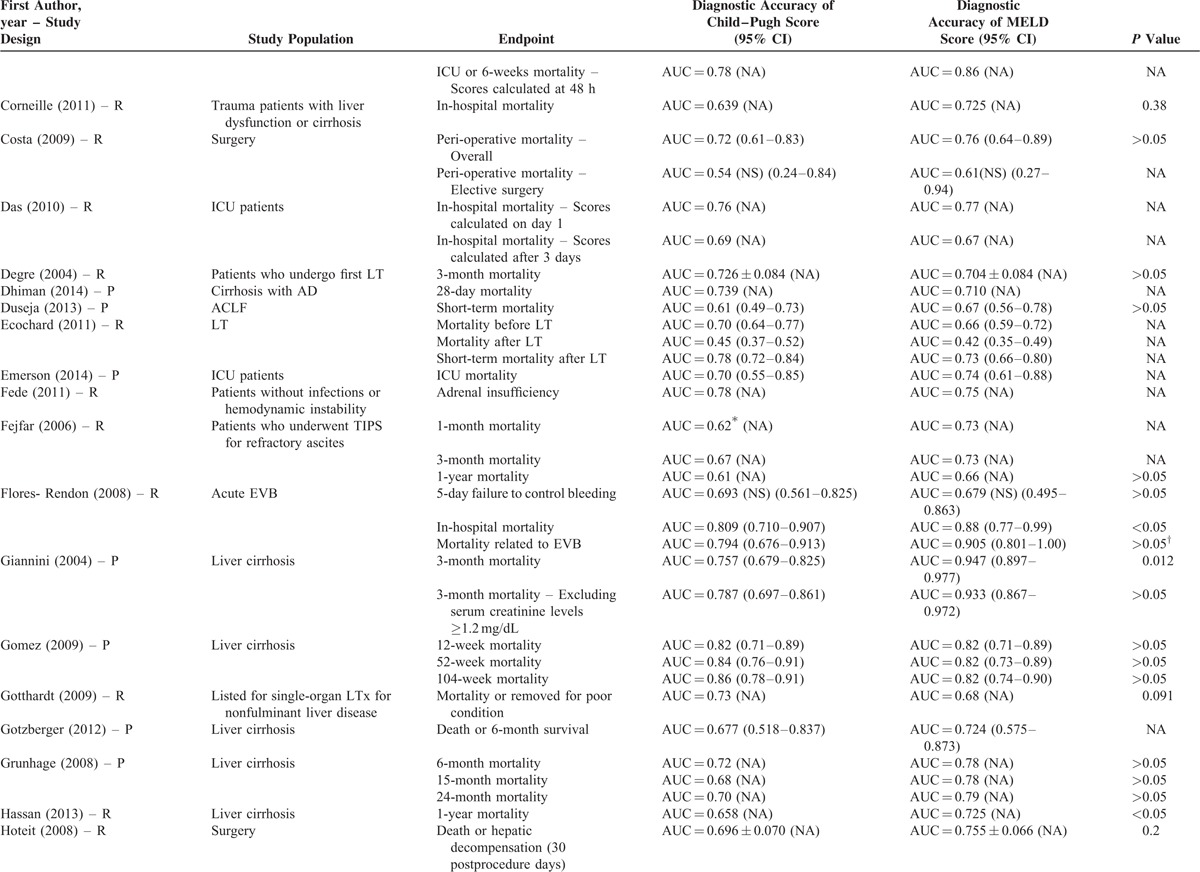
Results of Comparison Between MELD and Child–Pugh Score: An Overview of Studies

**TABLE 2 (Continued):**
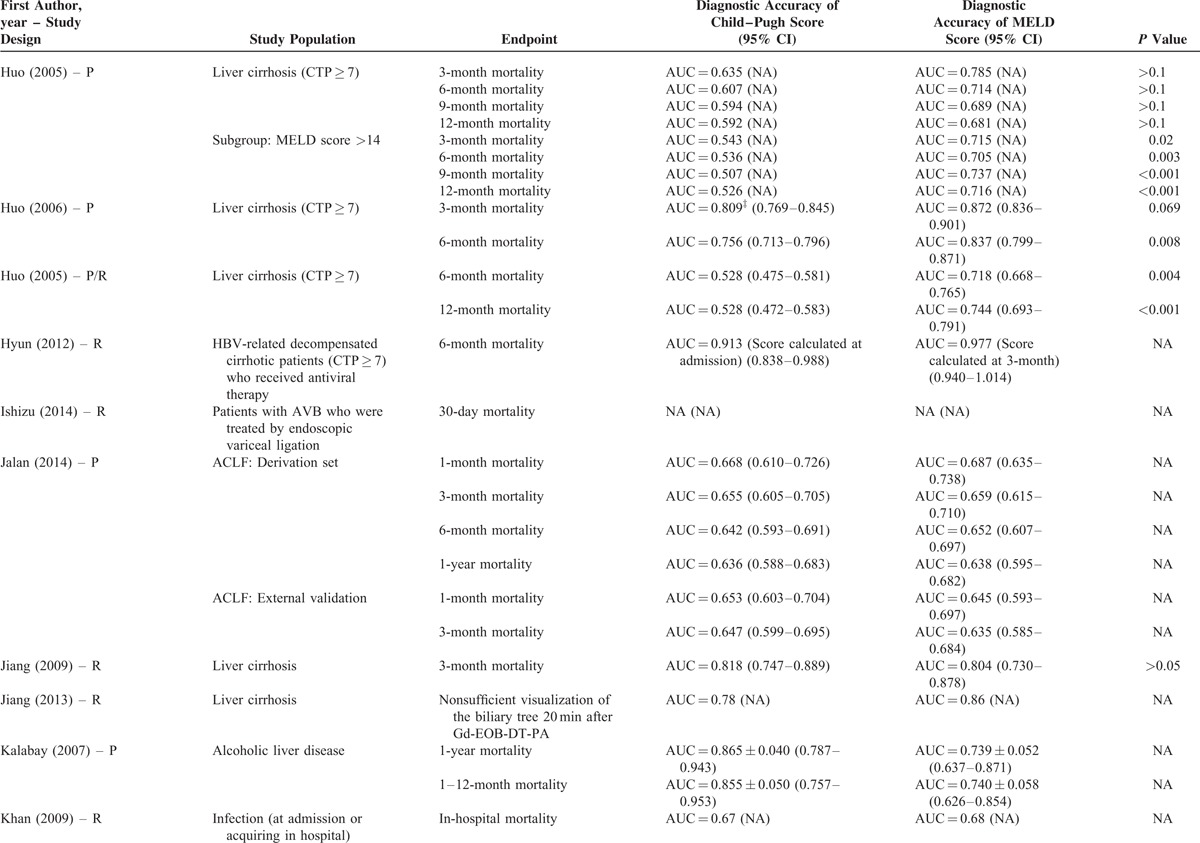
Results of Comparison Between MELD and Child–Pugh Score: An Overview of Studies

**TABLE 2 (Continued):**
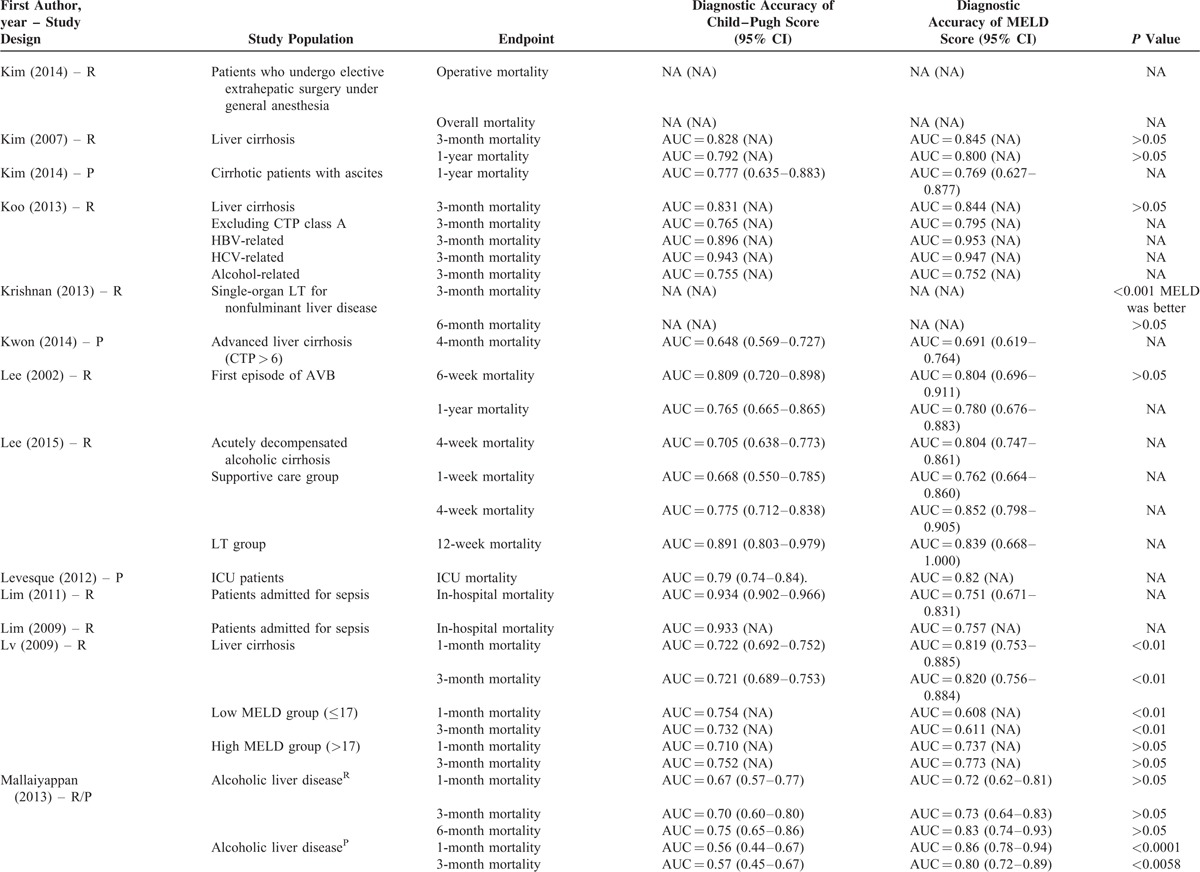
Results of Comparison Between MELD and Child–Pugh Score: An Overview of Studies

**TABLE 2 (Continued):**
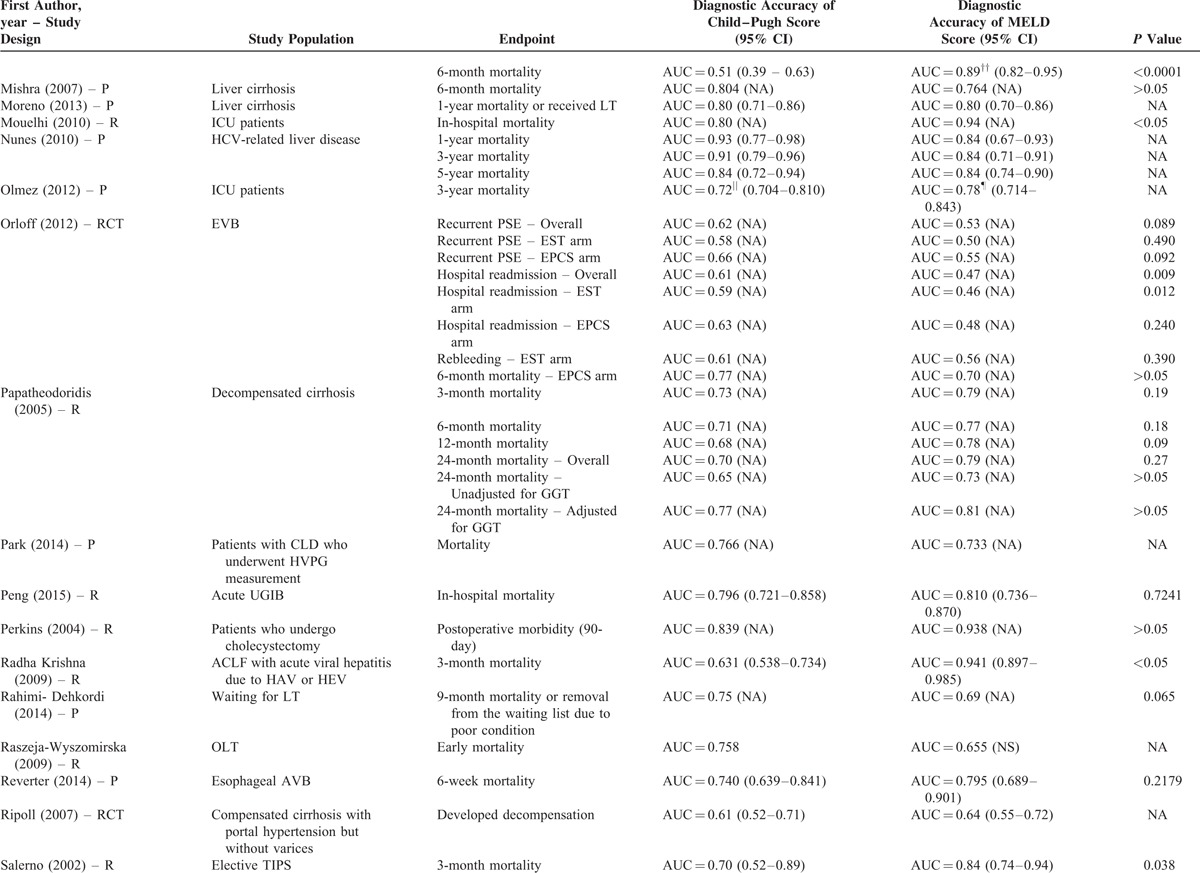
Results of Comparison Between MELD and Child–Pugh Score: An Overview of Studies

**TABLE 2 (Continued):**
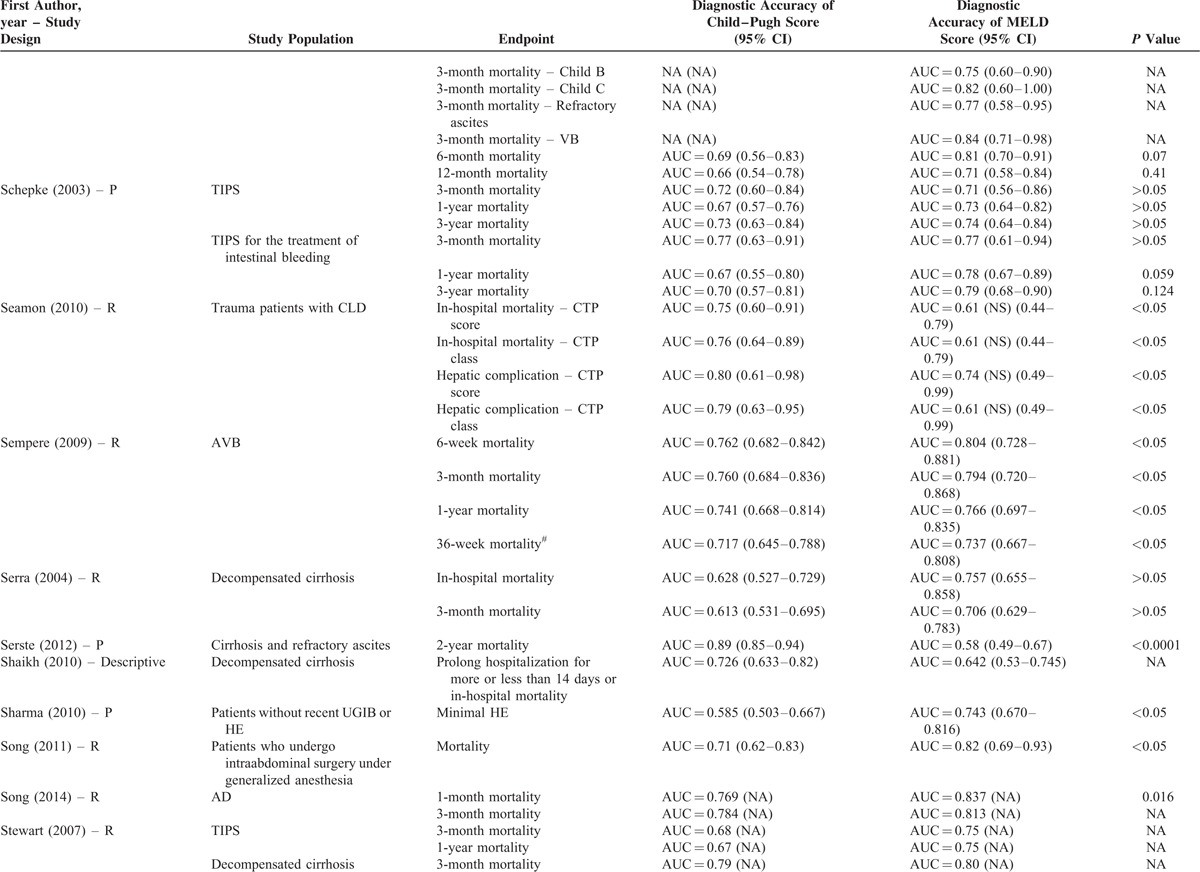
Results of Comparison Between MELD and Child–Pugh Score: An Overview of Studies

**TABLE 2 (Continued):**
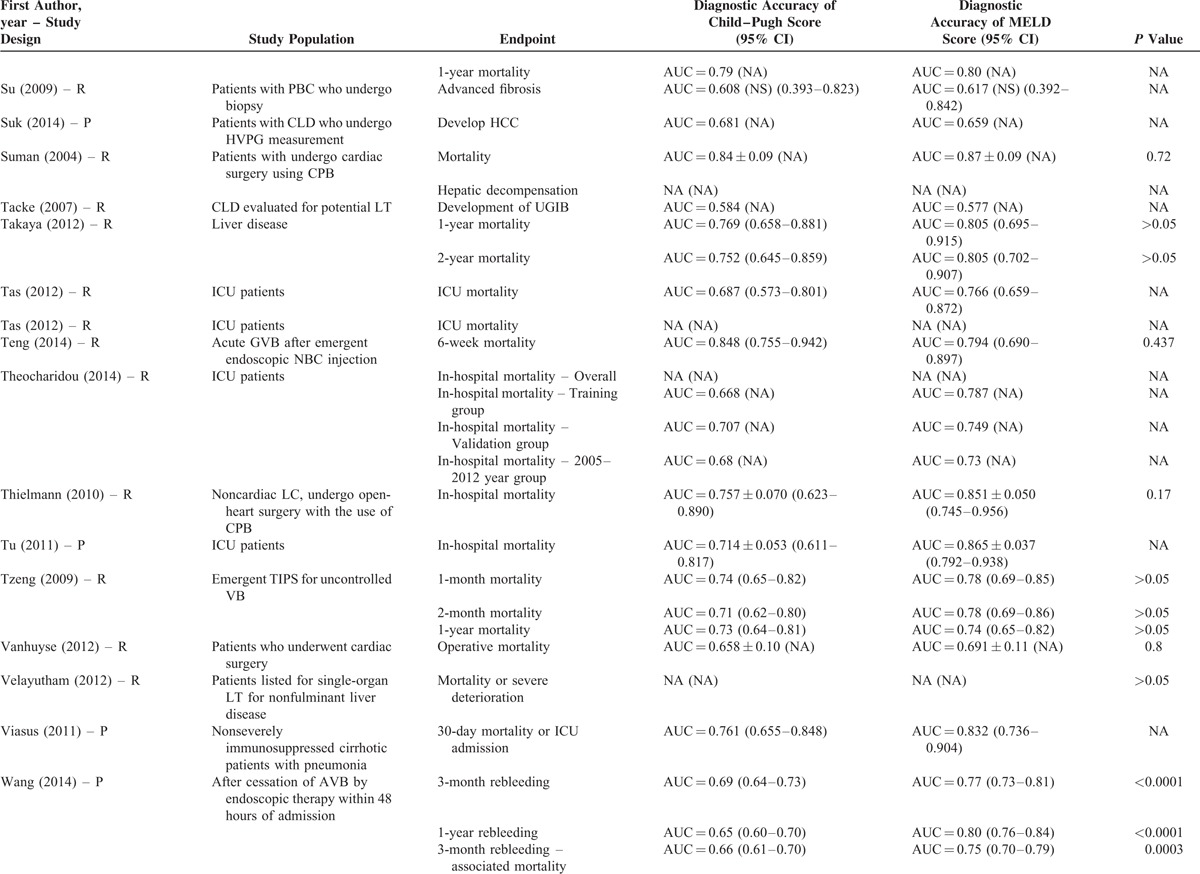
Results of Comparison Between MELD and Child–Pugh Score: An Overview of Studies

**TABLE 2 (Continued):**
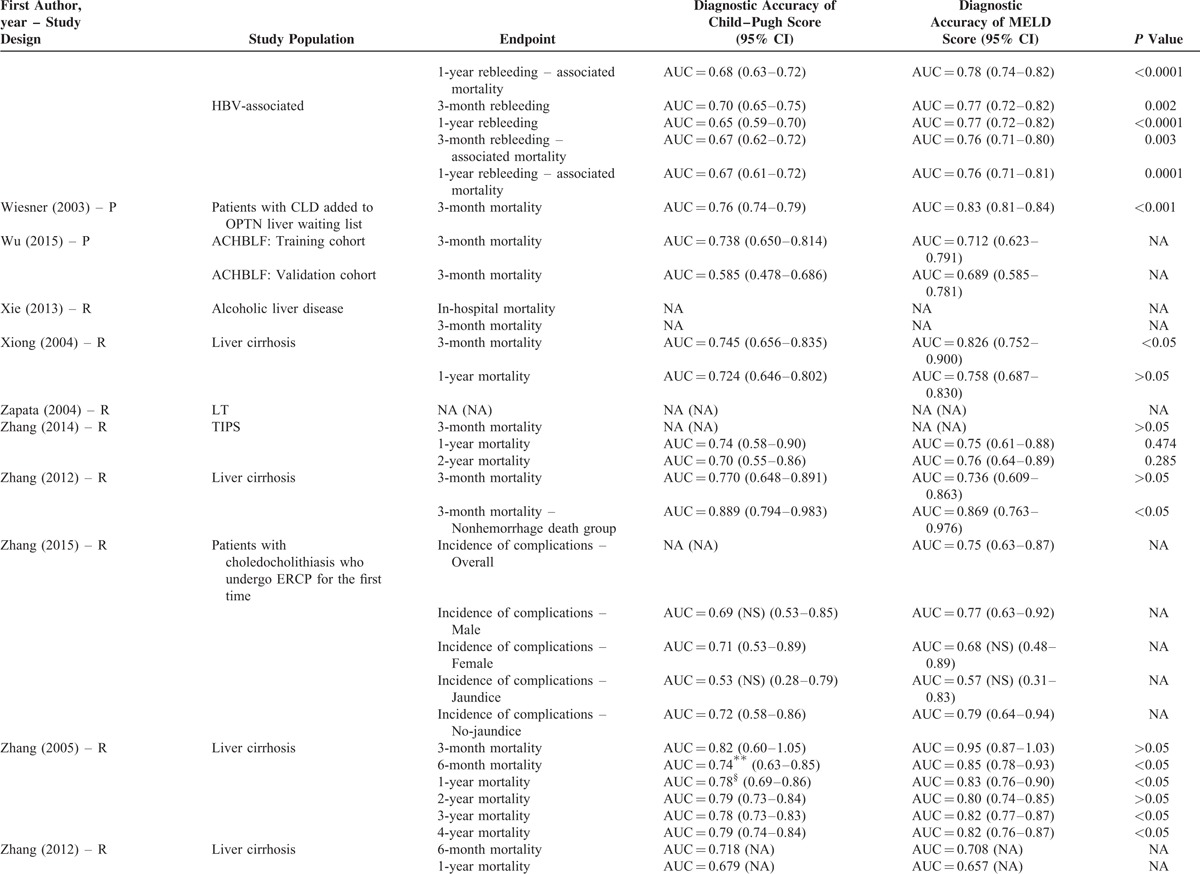
Results of Comparison Between MELD and Child–Pugh Score: An Overview of Studies

**TABLE 2 (Continued):**
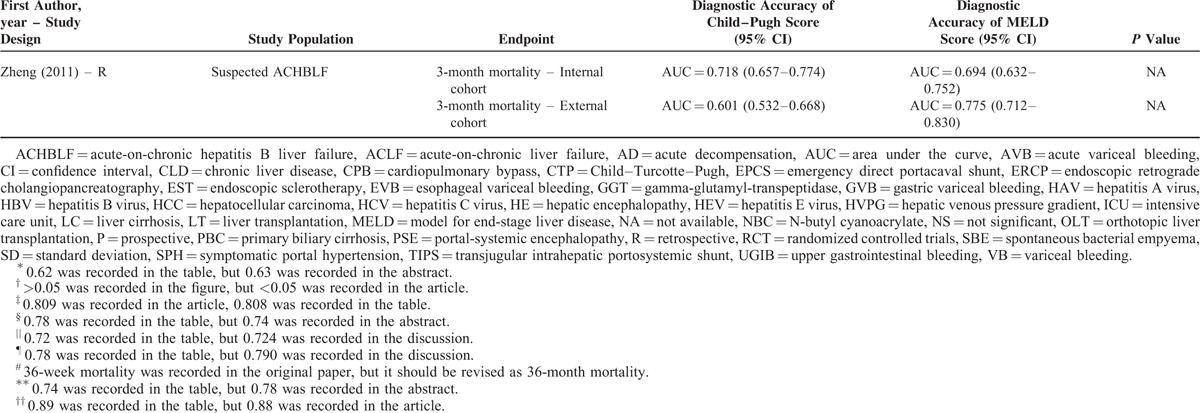
Results of Comparison Between MELD and Child–Pugh Score: An Overview of Studies

### Study Quality

The brief explanation of study quality was presented in Table 3  . As for the risk of bias, 48 and 71 studies had low and unclear risks in the term of patient selection, respectively; 119 studies had low risks in the term of index tests; 117 and 2 studies had low and unclear risks in the term of reference standard, respectively; 91 and 28 studies had low and unclear risks in the term of flow and timing, respectively. As for the applicability concerns, 94 and 25 studies had low and high concerns in the term of patient selection, respectively; 2, 1, and 116 studies had low, unclear, and high concerns in the term of index test, respectively; 1 and 118 studies had low and high concerns in the term of reference standard, respectively.

**TABLE 3:**
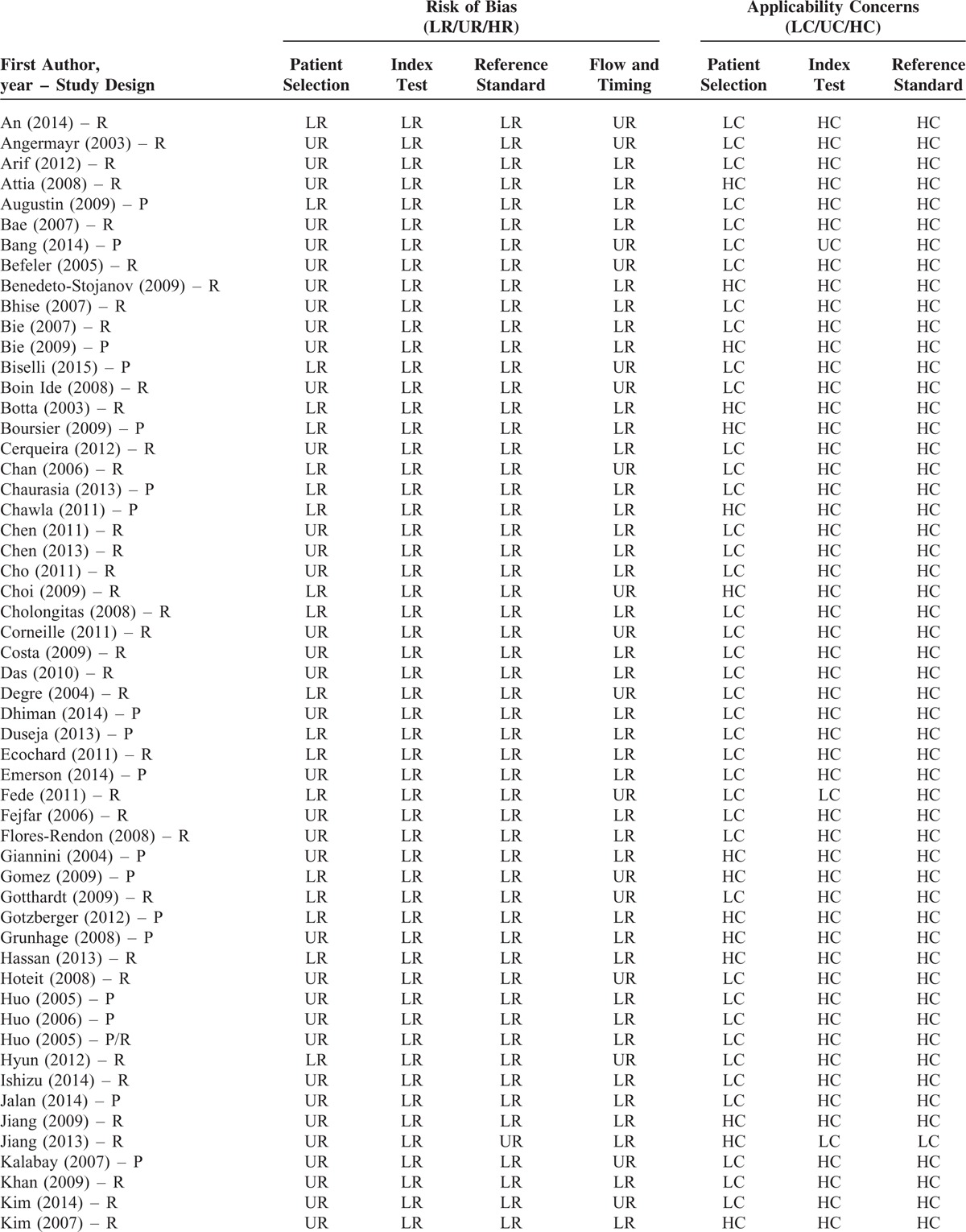
Quality Assessment

**TABLE 3 (Continued):**
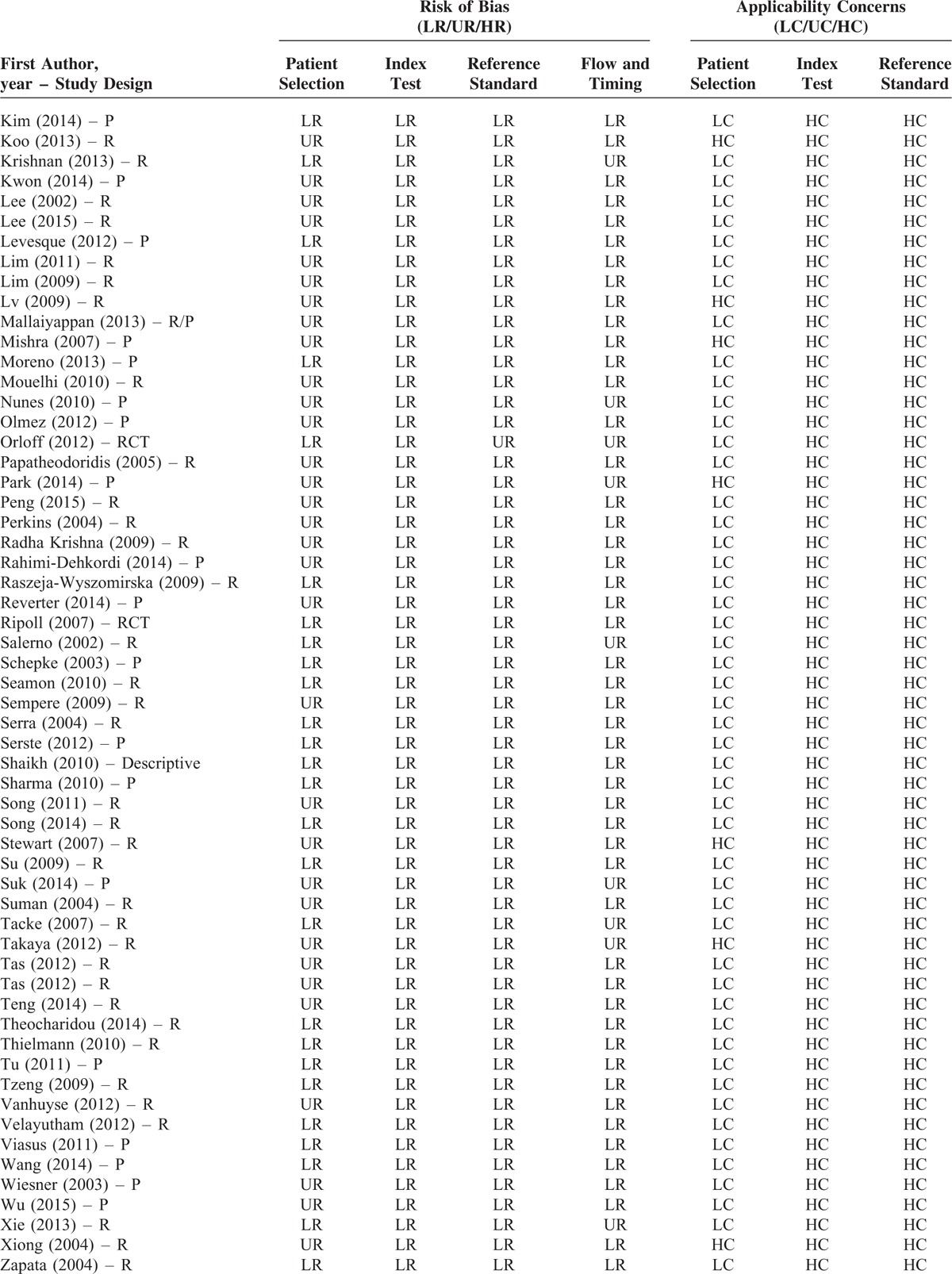
Quality Assessment

**TABLE 3 (Continued):**
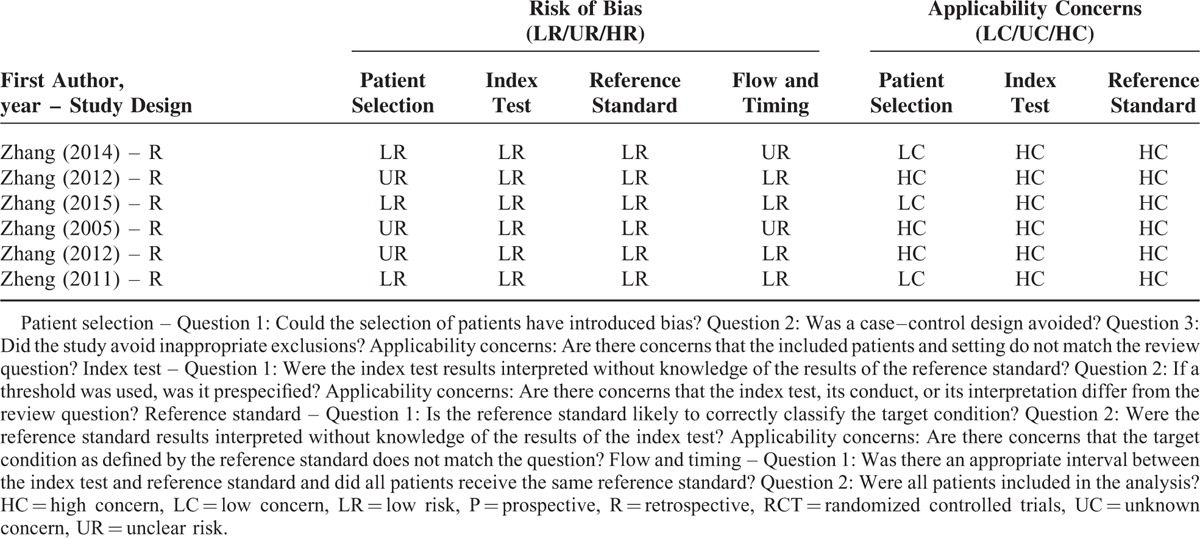
Quality Assessment

### Meta-Analysis

As for the meta-analysis, 77 papers were excluded,^12,14–16,20–23,26–31,33–39,41,43–47,49–51,53–55,57–60,63,64,66,68–73,75,78,79,81–83,85,86,88–90,92,93,95,96,99–101,103,105,106,113,114,118,120–124,126,128^ because 76 studies were lacking of relevant data^12,14–16,20–23,26–31,33–39,41,43–47,49–51,53–55,57–59,63,64,66,68–73,75,78,79,81–83,85–86,88–90,92,93,95,96,99–101,103,105,106,113,114,118,120–124,126,128^ and 1 study had the endpoint unrelated to the prognosis.^60^ Finally, 42 papers were included (Figure 1).^10,11,13,17–19,24,25,32,40,42,48,52,56,61–63,67,74,76,77,80,84,87,91,94,97,98,102,104,107–112,115–117,119,125,127^ Data extracted from these papers were summarized in Supplementary Table 1.

Meta-analyses were performed according to the clinical presentations, etiology of liver diseases, patients’ conditions, treatment options, and endpoints (Table 4).

**TABLE 4:**
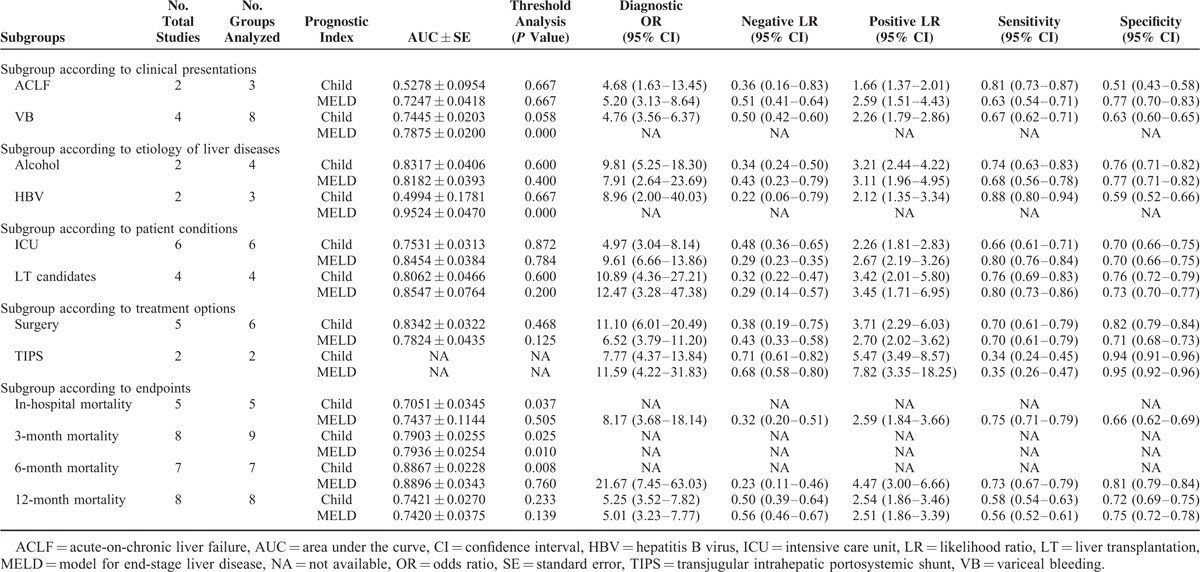
Results of Meta-Analyses

### Subgroup Analysis According to the Clinical Presentations

Two studies were eligible for the subgroup meta-analysis to compare the diagnostic accuracy of Child–Pugh versus MELD score in patients with ACLF.^40,119^ The mean AUSROC of MELD score was larger than that of Child–Pugh score. There was no statistically significant diagnostic threshold effect in the meta-analysis of Child–Pugh or MELD score. The 95%CIs of DORs, NLRs, and PLRs were overlapped between them. But the 95%CIs of sensitivities and specificities were not overlapped. Child–Pugh score had a higher summary sensitivity than MELD score, but MELD score had a higher summary specificity than Child–Pugh score.

Four studies were eligible for the subgroup meta-analysis to compare the diagnostic accuracy of Child–Pugh versus MELD score in patients with UGIB.^84,94,109,117^ The mean AUSROC of MELD score was larger than that of Child–Pugh score. There was a statistically significant diagnostic threshold effect in the meta-analysis of MELD score. Thus, DOR, NLR, PLR, sensitivity, or specificity of MELD score was not calculated.

### Subgroup Analysis According to the Etiology of Liver Diseases

Two studies were eligible for the subgroup meta-analysis to compare the diagnostic accuracy of Child–Pugh versus MELD score in patients with alcohol alone related liver cirrhosis.^19,61^ The mean AUSROC of Child–Pugh score was larger than that of MELD score. There was no statistically significant diagnostic threshold effect in the meta-analysis of Child–Pugh or MELD score. The 95%CIs of DORs, NLRs, PLRs, sensitivities, and specificities were overlapped between them.

Two studies were eligible for the subgroup meta-analysis to compare the diagnostic accuracy of Child–Pugh versus MELD score in patients with hepatitis B virus alone related liver cirrhosis.^56,119^ The mean AUSROC of MELD score was larger than that of Child–Pugh score. There was a statistically significant diagnostic threshold effect in the meta-analysis of MELD score. Thus, DOR, NLR, PLR, sensitivity, or specificity of MELD score was not calculated.

### Subgroup Analysis According to the Patients’ Conditions

Six studies were eligible for the subgroup meta-analysis to compare the diagnostic accuracy of Child–Pugh versus MELD score in patients admitted to ICU.^42,80,107,108,110,112^ The mean AUSROC of MELD score was larger than that of Child–Pugh score. There was no statistically significant diagnostic threshold effect in the meta-analysis of Child–Pugh or MELD score. The 95%CIs of DORs, PLRs, and specificities were overlapped between them. But the 95%CIs of NLRs and sensitivities were not overlapped. MELD score had a smaller summary NLR and a higher summary sensitivity than Child–Pugh score.

Four studies were eligible for the subgroup meta-analysis to compare the diagnostic accuracy of Child–Pugh versus MELD score in LT candidates.^48,67,87,115^ The mean AUSROC of MELD score was larger than that of Child–Pugh score. There was no statistically significant diagnostic threshold effect in the meta-analysis of Child–Pugh or MELD score. The 95%CIs of DORs, NLRs, PLRs, sensitivities, and specificities were overlapped between them.

### Subgroup Analysis According to the Treatment Options

Five studies were eligible for the subgroup meta-analysis to compare the diagnostic accuracy of Child–Pugh versus MELD score in patients who underwent surgery.^17,32,52,104,111^ The mean AUSROC of Child–Pugh score was larger than that of MELD score. There was no statistically significant diagnostic threshold effect in the meta-analysis of Child–Pugh or MELD score. The 95%CIs of DORs, NLRs, PLRs, and sensitivities were overlapped between them. But the 95%CIs of specificities were not overlapped. Child–Pugh score had a higher summary specificity than MELD score.

Two studies were eligible for the subgroup meta-analysis to compare the diagnostic accuracy of Child–Pugh versus MELD score in patients who underwent TIPS.^11,91^ Because only 2 comparisons were eligible for the subgroup meta-analysis, the mean AUSROCs of Child–Pugh and MELD scores could not be calculated. The 95%CIs of DORs, NLRs, PLRs, sensitivities, and specificities were overlapped between them.

### Subgroup Analysis According to the Endpoints

Five studies were eligible for the subgroup meta-analysis to compare the diagnostic accuracy of Child–Pugh versus MELD score for predicting the in-hospital mortality.^62,84,110–112^ The mean AUSROC of MELD score was larger than that of Child–Pugh score. There was a statistically significant diagnostic threshold effect in the meta-analysis of Child–Pugh score. DOR, NLR, PLR, sensitivity, or specificity of Child–Pugh score was not calculated.

Eight studies were eligible for the subgroup meta-analysis to compare the diagnostic accuracy of Child–Pugh versus MELD score for predicting the 3-month mortality.^11,19,32,74,91,94,117,119^ The mean AUSROC of MELD score was larger than that of Child–Pugh score. There were statistically significant diagnostic threshold effects in the meta-analyses of Child–Pugh and MELD scores. DORs, NLRs, PLRs, sensitivities, or specificities of Child–Pugh and MELD scores were not calculated.

Seven studies were eligible for the subgroup meta-analysis to compare the diagnostic accuracy of Child–Pugh versus MELD score for predicting the 6-month mortality.^19,24,25,56,67,76,127^ The mean AUSROC of MELD score was larger than that of Child–Pugh score. There was a statistically significant diagnostic threshold effect in the meta-analysis of Child–Pugh score. DOR, NLR, PLR, sensitivity, or specificity of Child–Pugh score was not calculated.

Eight studies were eligible for the subgroup meta-analysis to compare the diagnostic accuracy of Child–Pugh versus MELD score for predicting the 12-month mortality.^13,24,61,65,77,94,117,127^ The mean AUSROC of Child–Pugh score was larger than that of MELD score. There was no statistically significant diagnostic threshold effect in the meta-analysis of Child–Pugh or MELD score. The 95%CIs of DORs, NLRs, PLRs, sensitivities, and specificities were overlapped between them.

## DISCUSSION

To our knowledge, this is the most comprehensive review to evaluate the diagnostic accuracy of Child–Pugh and MELD scores in patients with liver cirrhosis. Indeed, several previous narrative reviews regarding their prognostic values had been published by top experts.^129–131^ By comparison, our study employed a systematic search strategy to maximize the number of relevant papers. Several additional strengths included: the study and patient characteristics were systematically analyzed; the study quality was carefully evaluated; the clinical significance of Child–Pugh and MELD scores was further subdivided according to the different study population; and the meta-analysis was employed to synthesize the statistical results. Some remarkable findings should be summarized as follows.

First, in patients with ACLF, Child–Pugh score had a significantly higher sensitivity than MELD score, because the 95%CIs were not overlapped among them and the lower limit of 95%CI of Child–Pugh score was higher than the upper limit of 95%CI of MELD score (0.73 > 0.71); by contrast, MELD score had a significantly higher specificity than Child–Pugh score, because the 95%CIs were not overlapped among them and the lower limit of 95%CI of MELD score was higher than the upper limit of 95%CI of Child–Pugh score (0.70 > 0.58). These findings suggested that Child–Pugh score might have a better discriminative ability to predict the probability of developing some endpoint events in patients with ACLF, and that MELD score might have a better discriminative ability to predict the probability of free of developing some endpoint events in such patients.

Second, in patients admitted to ICU, MELD score had a significantly smaller NLR than Child–Pugh score, because the 95%CIs were not overlapped among them and the upper limit of 95%CI of MELD score was smaller than the lower limit of 95%CI of Child–Pugh score (0.35<0.36). MELD score also had a significantly higher sensitivity than Child–Pugh score, because the 95%CIs were not overlapped among them and the lower limit of 95%CI of MELD score was higher than the upper limit of 95%CI of Child–Pugh score (0.76 > 0.71). These findings suggested that MELD score might have a better discriminative ability to predict the probability of developing some endpoint events in such patients.

Third, in patients undergoing surgery, Child–Pugh score had a significantly higher specificity than MELD score, because the 95%CIs were not overlapped among them and the lower limit of 95%CI of Child–Pugh score was higher than the upper limit of 95%CI of MELD score (0.79 > 0.73). These findings suggested that Child–Pugh score might have a better discriminative ability to predict the probability of free of developing some endpoint events in such patients.

Fourth, Child–Pugh and MELD scores had statistically similar discriminative abilities in some subgroups (i.e., patients with alcohol alone related liver cirrhosis, LT candidates, patients undergoing TIPS, and 12-month mortality as the endpoint).

Fifth, because of statistically significant diagnostic threshold effects, DORs, NLRs, PLRs, sensitivities, or specificities could not be compared in some subgroups (i.e., patients with acute gastrointestinal bleeding, patients with hepatitis B virus alone related liver cirrhosis, in-hospital mortality as the endpoint, 3-month mortality as the endpoint, and 6-month mortality as the endpoint).

Our study had 2 major limitations. First, although a great number of papers were included in the systematic review, not all included studies were eligible for our meta-analysis. Additionally, in some subgroup analyses, DORs, NLRs, PLRs, sensitivities, or specificities were not available. Thus, the combination of data from some selected papers could result in the potential bias. Second, the cut-off values of Child–Pugh and MELD scores for the assessment of prognosis were different among included studies. Therefore, we could not obtain any accurate thresholds for identifying the high-risk or low-risk patients.

In conclusion, we provided an overview regarding the comparison of Child–Pugh and MELD scores for the assessment of prognosis in liver cirrhosis. Both of them had similar prognostic significance in most of cases. However, given their distinctive benefits for some specific conditions, further studies might be necessary to clarify the candidates who should use Child–Pugh or MELD score for the assessment of prognosis and the timing when we should use Child–Pugh or MELD score for the assessment of prognosis. New scores should also be proposed to more accurately assess the prognosis of patients with liver disease based on prospective studies.

## Supplementary Material

Supplemental Digital Content
