# Supplementary material for: Child–Pugh Versus MELD Score for the Assessment of Prognosis in Liver Cirrhosis: A Systematic Review and Meta-Analysis of Observational Studies
Source: Medicine (Baltimore). 2016 Mar 3;95(8):e2877. doi: 10.1097/MD.0000000000002877 (PMC4779019; doi:10.1097/MD.0000000000002877)
Supplement: Supplemental Digital Content [file medi-95-e2877-s001.doc]

**Supplementary Table 1. Pooled data: an overview of 42 studies included in the meta-analysis**

| **First  author  (Year) - study design** | **No. total Pts / No. Pts analyzed** | **Study population** | **Endpoint** |  | **Child-Pugh score** | | | | | | | | | | |  | **MELD score** | | | | | | | | | |
| --- | --- | --- | --- | --- | --- | --- | --- | --- | --- | --- | --- | --- | --- | --- | --- | --- | --- | --- | --- | --- | --- | --- | --- | --- | --- | --- |
|  |  |  |  |  | **a+c** | **Cut- off** | **TP (a)** | **FP  (b)** | **FN  (c)** | | **TN  (d)** | **Sen. (%)** | **Spe.  (%)** | **PPV  (%)** | **NPV  (%)** |  | **a+c** | **Cut- off** | **TP (a)** | **FP  (b)** | **FN  (c)** | **TN  (d)** | **Sen. (%)** | **Spe.  (%)** | **PPV  (%)** | **NPV  (%)** |
| An (2014) - R | 17/14 | Recurrent HE after embolisation | 2-year  mortality |  | 4 | 10 | 4 | 1 | 0 | | 9 | 100% | 91% | NA | NA |  | 4 | 15 | 4 | 0 | 0 | 10 | 100% | 100% | NA | NA |
| Angermayr (2003) - R | 475/475 | Elective TIPS | 3-month  mortality |  | 75 | 11 | 25 | 24 | 50 | 376 | | 33% | 94% | NA | NA |  | 75 | 14 | 26 | 24 | 49 | 376 | 34% | 94% | NA | NA |
| Attia (2008) - R | 172/172 | Black African patients with cirrhosis | 12-month  mortality |  | 54 | 10 | 38 | 60 | 16 | | 58 | 70.4%^*^ | 49.20% | 38.80% | 78.40% |  | 54 | 21 | 26 | 36 | 28 | 82 | 48.20% | 69.50% | 41.20% | 74.60% |
| Befeler (2005) - R | 53/53 | Patients who undergo abdominal surgery | Poor outcome |  | 13 | Child C | 3 | 2 | 10 | | 38 | 23% | 95% | 60% | 78% |  | 13 | 14 | 10 | 8 | 3 | 32 | 77% | 80% | 56% | 91% |
| Benedeto- Stojanov (2009) - R | 100/100 | Patients with complications of liver disease | 15-month  mortality |  | 22 | 8 | 21 | 14 | 1 | | 64 | 95.45% | 82.05% | NA | NA |  | 22 | 6 | 21 | 36 | 1 | 42 | 95.45% | 53.84% | NA | NA |

| Bhise (2007) - R | 79/79 | Alcoholic cirrhotic patients | 3-month mortality |  | 7 | 8.5 | 6 | 19 | 1 | 53 | 85% | 73% | NA | NA |  | 7 | 14 | 6 | 14 | 1 | 58 | 86% | 80% | NA | NA |
| --- | --- | --- | --- | --- | --- | --- | --- | --- | --- | --- | --- | --- | --- | --- | --- | --- | --- | --- | --- | --- | --- | --- | --- | --- | --- |
|  |  |  | 6-month  mortality |  | 13 | 8.5 | 9 | 16 | 4 | 50 | 69% | 75% | NA | NA |  | 13 | 12 | 12 | 13 | 1 | 53 | 92% | 80% | NA | NA |
| Botta (2003) - R | 129/129 | Liver cirrhosis | 6-month mortality |  | 12 | 8 | 11 | 33 | 1 | 84 | 92% | 72% | NA | NA |  | 12 | 8 | 9 | 37 | 3 | 80 | 75% | 68% | NA | NA |
|  |  |  | 1-year mortality |  | 31 | 8 | 19 | 24 | 12 | 74 | 61% | 75% | NA | NA |  | 31 | 6 | 27 | 57 | 4 | 41 | 87% | 42% | NA | NA |
| Boursier (2009) - P | 308/308 | Liver cirrhosis | 6-month mortality |  | 45 | 10 | 38 | 45 | 7 | 218 | 84.1% | 82.7% | 45.1% | 96.8% |  | 45 | 16.8 | 34 | 39 | 11 | 224 | 75.6% | 85.1% | 46.6% | 95.3% |
|  | 154/154 | Decompensated cirrhosis | 6-month mortality |  | 42 | 10 | 38 | 45 | 4 | 67 | 90.2% | 59.8% | 45.1% | 94.4% |  | 42 | 17.9 | 30 | 25 | 12 | 87 | 71.4% | 77.7% | 54.5% | 87.9% |
| Cho (2011) - R | 490/490 | Patients who undergo non-hepatic surgery under general anesthesia | 1-month mortality |  | 18 | Child B,C | 14 | 90 | 4 | 382 | 78% | 81% | 13% | 99% |  | 18 | 10 | 11 | 151 | 7 | 321 | 61% | 68% | 7% | 98% |
|  |  |  | 3-month mortality |  | 35 | Child B,C | 27 | 77 | 8 | 378 | 77% | 83% | 26% | 98% |  | 35 | 10 | 23 | 136 | 12 | 319 | 66% | 70% | 14% | 96% |
| Duseja (2013) - P | 100/100 | ACLF | Short-term mortality |  | 53 | 12 | 37 | 22 | 16 | 25 | 69.8% | 53.1% | 62.7% | 60.9% |  | 53 | 29 | 38 | 20 | 15 | 27 | 71.6% | 57.4% | 65.5% | 64.2% |
| Emerson (2014) - P | 59/59 | ICU patients | ICU mortality |  | 18 | 9.5 | 11 | 16 | 7 | 25 | 61% | 62% | NA | NA |  | 18 | 18 | 15 | 14 | 3 | 27 | 83% | 67% | NA | NA |
| Gotthardt (2009) - R | 268/168 | Listed for single-organ LTx for nonfulminant liver disease | Mortality or removed for poor condition |  | 29 | 9 | 20 | 41 | 9 | 98 | 69.0% | 70.5% | NA | NA |  | 29 | 14.4 | 18 | 38 | 11 | 101 | 62.1% | 72.7% | NA | NA |
| Hoteit (2008) - R | 195/57 | Surgery | Death or hepatic decompensation |  | 21 | 9 | 17 | 19 | 4 | 17 | 81% | 47% | NA | NA |  | 21 | 17 | 15 | 10 | 6 | 26 | 71% | 72% | NA | NA |
| Hyun (2012) - R | 86/83 | HBV-related decompensated cirrhotic patients who received antiviral therapy | 6-month  mortality |  | 5 | 11 | 5 | 19 | 0 | 59 | 100% | 75.6% | NA | NA |  | 5 | 17.5 | 5 | 7 | 0 | 71 | 100% | 91% | NA | NA |
| Kalabay (2007) - P | 93/89 | Alcoholic liver disease | 1-year  mortality |  | 37 | 10 | 28 | 9 | 9 | 43 | 75.3% | 82.7% | NA | NA |  | 37 | 20 | 22 | 14 | 15 | 38 | 59.5% | 72.9% | NA | NA |
|  | 93/75 | Alcoholic liver disease | 1-12-month  mortality |  | 23 | 10 | 16 | 13 | 7 | 39 | 67.5% | 75.5% | NA | NA |  | 23 | 20 | 14 | 15 | 9 | 37 | 58.7% | 72.0% | NA | NA |
| Kim (2014) - P | 65/65 | Cirrhotic patients with ascite | 1-year mortality |  | 10 | 9 | 8 | 16 | 2 | 39 | 80.0% | 71.8% | NA | NA |  | 10 | 14 | 8 | 13 | 2 | 42 | 80.0% | 76.9% | NA | NA |
| Khan (2009) - R | 530/530 | Infection | In-hospital  mortality |  | 186 | 11 | 136 | 220 | 50 | 124 | 73% | 36.0% | NA | NA |  | 186 | 22 | 126 | 141 | 60 | 203 | 68% | 59% | NA | NA |
| Krishnan (2013) - R | 216/216 | Single-organ LT for nonfulminant liver disease | 6-month  mortality |  | 56 | NA | 47 | 17 | 9 | 143 | 83.9% | 89.5% | NA | NA |  | 56 | NA | 50 | 14 | 6 | 146 | 88.6% | 91.0% | NA | NA |
| Lv (2009) - R | 256/256 | Liver cirrhosis | 1-month  mortality |  | 61 | 9.5 | 42 | 60 | 19 | 135 | 68.9% | 69.2% | NA | NA |  | 61 | 17 | 44 | 33 | 17 | 162 | 72.1% | 83.1% | NA | NA |
|  |  |  | 3-month  mortality |  | 76 | 9.5 | 51 | 55 | 25 | 125 | 67.1% | 69.4% | NA | NA |  | 76 | 17.5 | 54 | 23 | 22 | 157 | 71.1% | 87.2% | NA | NA |
| Mishra (2007) - P | 76/76 | Liver cirrhosis | 6-month  mortality |  | 16 | 8 | 15 | 27 | 1 | 33 | 94% | 55% | NA | NA |  | 16 | 11 | 15 | 17 | 1 | 43 | 94% | 71% | NA | NA |
| Moreno (2013) - P | 125/125 | Liver cirrhosis | 1-year  mortality or received LT |  | 36 | 10 | 28 | 25 | 8 | 64 | 77.7% | 71.9% | 52.8% | 88.8% |  | 36 | 24 | 21 | 11 | 15 | 78 | 58.3% | 87.6% | 65.6% | 83.8% |
| Olmez (2012) - P | 201/201 | ICU patients | 3-year  mortality |  | 84 | 10.5 | 58 | 39 | 26 | 78 | 69% | 67% | NA | NA |  | 84 | 19.5 | 67 | 42 | 17 | 75 | 79.5% | 64% | NA | NA |
| Peng (2015) - R | 145/145 | Acute UGIB | In-hospital  mortality |  | 11 | 9 | 7 | 28 | 4 | 106 | 63.6% | 79.1% | NA | NA |  | 11 | 12 | 9 | 37 | 2 | 97 | 83.6% | 72.7% | NA | NA |
| Rahimi- Dehkordi (2014) - P | 257/257 | Waiting for LT | 9-month  mortality or poor condition |  | 31 | 8 | 23 | 75 | 8 | 151 | 74% | 67% | NA | NA |  | 31 | 13.7 | 23 | 95 | 8 | 131 | 74% | 58% | NA | NA |
| Salerno (2002) - R | 140/138 | Elective TIPS | 3-month  mortality |  | 11 | 10 | 4 | 9 | 7 | 118 | 36% | 93% | 30% | 94% |  | 11 | 18 | 5 | 4 | 6 | 123 | 45% | 97% | 55% | 95% |

| Sempere (2009) - R | 201/201 | AVB | 6-week  mortality |  | 46 | 10 | 29 | 40 | 17 | 115 | 63.0% | 74.2% | 42.0% | 87.1% |  | 46 | 18 | 26 | 18 | 20 | 137 | 56.5% | 88.4% | 59.1% | 87.3% |
| --- | --- | --- | --- | --- | --- | --- | --- | --- | --- | --- | --- | --- | --- | --- | --- | --- | --- | --- | --- | --- | --- | --- | --- | --- | --- |
|  |  |  | 3-month  mortality |  | 50 | 10 | 30 | 39 | 20 | 112 | 60.0% | 74.2% | 43.5% | 84.8% |  | 50 | 18 | 27 | 17 | 23 | 134 | 54.0% | 88.7% | 61.3% | 85.3% |
|  |  |  | 12-month  mortality |  | 69 | 10 | 41 | 28 | 28 | 104 | 59.4% | 78.8% | 59.4% | 78.8% |  | 69 | 18 | 33 | 11 | 36 | 121 | 47.8% | 91.6% | 75.0% | 77.1% |
|  |  |  | 36-week  mortality ^$^ |  | 80 | 10 | 43 | 26 | 37 | 95 | 53.7% | 78.5% | 62.3% | 71.9% |  | 80 | 18 | 34 | 10 | 46 | 111 | 42.5% | 91.7% | 77.2% | 70.7% |
| Shaikh (2010) - Descriptive | 110/110 | Decompensated cirrhosis | Prolong hospitalization ≥14 days or in-hospital mortality |  | 56 | NA | 44 | 20 | 12 | 34 | 77.7% | 63.0% | 18.9%^a^ | 96.2%^b^ |  | 56 | NA | 25 | 13 | 31 | 41 | 44% | 75% | 16.48%^c^ | 92.3%^d^ |
| Sharma (2010) - P | 200/200 | Patients without recent UGIB or HE | Minimal  HE |  | 82 | 7.5 | 52 | 63 | 30 | 55 | 63.4% | 47.0% | NA | NA |  | 82 | 15.5 | 61 | 29 | 21 | 89 | 74.4% | 75.2% | NA | NA |
| Su (2009) - R | 46/46 | Patients with PBC who undergo biopsy | Advanced  fibrosis |  | 11 | 8 | 4 | 0 | 7 | 35 | 36.4% | 100% | 100% | 83.3% |  | 11 | 13 | 4 | 2 | 7 | 33 | 36.4% | 94.3% | 66.7% | 82.5% |
| Suman (2004) - R | 44/44 | Patients with undergo cardiac surgery using CPB | Mortality |  | 7 | 7 | 6 | 3 | 1 | 34 | 86% | 92% | 67% | 97% |  | 7 | 13 | 5 | 4 | 2 | 33 | 71% | 89% | 56% | 94% |
|  |  |  | Hepatic decompensation |  | 12 | 7 | 8 | 1 | 4 | 31 | 66% | 97% | 89% | 88% |  | 12 | 13 | 8 | 1 | 4 | 31 | 67% | 97% | 89% | 88% |
| Tas (2012) - R | 90/90 | ICU patients | ICU  mortality |  | 37 | 10.5 | 23 | 17 | 14 | 36 | 63% | 68%^k^ | 53%^j^ | 78% |  | 37 | 19.5 | 28 | 18 | 9 | 35 | 75% | 66% | 60% | 79% |
| Tas (2012) - R | 106/106 | ICU patients | ICU  mortality |  | 36 | 9.5 | 33 | 17 | 3 | 53 | 91.7% | 75.7% | 66.0% | 94.6% |  | 36 | 19 | 30 | 12 | 6 | 58 | 83.3% | 82.9% | 71.4% | 90.6% |
| Teng (2014) - R | 132/132 | Acute GVB after emergent endoscopic NBC injection | 6-week  mortality |  | 22 | 9 | 20 | 34 | 2 | 76 | 90.9% | 69.1% | 37.0% | 97.4% |  | 22 | 18 | 15 | 21 | 7 | 89 | 68.2% | 80.9% | 39.5% | 92.6% |
| Theocharidou (2014) - R | 158/158 | ICU patients | In-hospital  mortality |  | 83 | 12.5 | 39 | 13 | 44 | 62 | 46.9% | 83.3% | 79.0% | 50.0% |  | 83 | 21 | 63 | 21 | 20 | 54 | 76.5% | 71.9% | 78.0% | 71.0% |
| Thielmann (2010) - R | 57/57 | Non-cardiac liver cirrhosis, undergo open-heart surgery using CPB | In-hospital  mortality |  | 17 | Class 1.5 | 11 | 7 | 6 | 33 | 64.7% | 82.5% | NA | NA |  | 17 | 13.5 | 14 | 9 | 3 | 31 | 82.0% | 78.5% | NA | NA |
| Tu (2011) - P | 202/202 | ICU patients | In-hospital  mortality |  | 121 | 11 | 88 | 28 | 33 | 53 | 73% | 66% | NA | NA |  | 121 | 24 | 102 | 23 | 19 | 58 | 84% | 72% | NA | NA |
| Velayutham (2012) - R | 210/210 | Patients listed for single-organ LT for nonfulminant liver disease | Mortality or severe deterioration (short-term) |  | 48 | NA | 35 | 33 | 13 | 129 | 73.9% | 79.5% | NA | NA |  | 48 | NA | 40 | 36 | 8 | 126 | 82.6% | 78.0% | NA | NA |
| Viasus (2011) - P | 90/ 90 | Nonseverely immunosuppressed cirrhotic patients with pneumonia | 30-day mortality or ICU admission |  | 18 | 11 | 9 | 1 | 9 | 71 | 50.0% | 98.5% | NA | NA |  | 18 | 20 | 12 | 3 | 6 | 69 | 64.7% | 95.5% | NA | NA |
| Wang (2014) - P | 429/429 | After cessation of AVB by endoscopic therapy within 48h | 3-month  rebleeding |  | 97 | 10 | 81 | 183 | 16 | 149 | 84% | 45% | 84%^e^ | 45.4%^f^ |  | 97 | 11 | 66 | 76 | 31 | 256 | 68% | 77% | 91.1%^g^ | 41%^h^ |
|  |  |  | 1-year  rebleeding |  | 206 | 9 | 152 | 111 | 54 | 112 | 74% | 50% | 61.2% | 63.4% |  | 206 | 10 | 159 | 62 | 47 | 161 | 77% | 72% | 74.7% | 74.0% |
|  |  |  | 3-month  rebleeding-  associated mortality |  | 53 | 11 | 49 | 226 | 4 | 150 | 92% | 40% | 91.6% | 42.0% |  | 53 | 14 | 44 | 169 | 9 | 207 | 83% | 55% | 92.8% | 30.5% |
|  |  |  | 1-year  rebleeding-  associated mortality |  | 98 | 10 | 68 | 139 | 30 | 192 | 69% | 58% | 84.7% | 35.4% |  | 98 | 11 | 66 | 83 | 32 | 248 | 67% | 75% | 89.8% | 39.9% |
| Wu (2015) - P | 121/121 | ACHBLF: Training cohort | 3-month  mortality |  | 51 | 10 | 48 | 34 | 3 | 36 | 93.6% | 51.5% | 55.0% | 92.7% |  | 51 | 29.4 | 29 | 11 | 22 | 59 | 57.5% | 83.8% | 69.2% | 75.6% |
|  | 93/93 | ACHBLF: Validation cohort | 3-month  mortality |  | 30 | 10 | 23 | 33 | 7 | 30 | 75.9% | 46.9% | 39.3% | 81.1% |  | 30 | 24.6 | 17 | 11 | 13 | 52 | 55.2% | 82.8% | 59.3% | 80.3% |

| Zhang  (2015) - R | 77/22 | Patients with choledocholithiasis who undergo ERCP for the first time (No-jaundice) | Incidence of complications |  | 2 | 8.5 | 1 | 3 | 1 | 17 | 50.0% | 82.9% | NA | NA |  | 2 | 11.5 | 2 | 7 | 0 | 13 | 78.6% | 65.9% | NA | NA |
| --- | --- | --- | --- | --- | --- | --- | --- | --- | --- | --- | --- | --- | --- | --- | --- | --- | --- | --- | --- | --- | --- | --- | --- | --- | --- |
| Zhang (2012) - R | 435/435 | Liver cirrhosis | 6-month  mortality |  | 107 | 9.5 | 45 | 29 | 62 | 299 | 42.1% | 91.2% | NA | NA |  | 107 | 12.5 | 61 | 72 | 46 | 256 | 57.0% | 78.0% | NA | NA |
|  |  |  | 1-year  mortality |  | 150 | 9.5 | 52 | 22 | 98 | 263 | 34.7% | 92.3% | NA | NA |  | 150 | 12.5 | 69 | 64 | 81 | 221 | 46.0% | 77.5% | NA | NA |
| **Abbreviations:** ACLF, acute-on-chronic liver failure; ACHBLF, acute-on-chronic hepatitis B liver failure; AVB, acute variceal bleeding; CPB, cardiopulmonary bypass; ERCP, endoscopic retrograde cholangiopancreatography; FN, false negative; FP, false positive; GVB, gastric variceal bleeding; HBV, hepatitis B virus; HE, hepatic encephalopathy; ICU, intensive care unit; LT, liver transplantation; MELD, model for end-stage liver disease; NA, not available; NBC, N-butyl cyanoacrylate; NPV, negative predictive value; P, prospective; PBC, primary biliary cirrhosis; PPV, positive predictive value; R, retrospective; Sen., sensitivity; Spe., specificity; TP, true positive; TIPS, transjugular intrahepatic portosystemic shunt; TN, true negative; UGIB, upper gastrointestinal bleeding. | | | | | | | | | | | | | | | | | | | | | | | | | |
| **Notes:** *, 70.4% was recorded in the table,but 74% was recorded in the results. $, 36-week mortality was recorded in the original paper, but it should be revised as 36-month mortality. k, 68% was recorded in the original paper, but it should be revised as 62%. j, 53% was recorded in the original paper, but it should be revised as 57.5%. a, 18.9% was recorded in the original paper, but it should be revised as 68.8%.  b, 96.2% was recorded in the original paper, but it should be revised as 73.9%. c, 16.48% was recorded in the original paper, but it should be revised as 65.8%. d, 92.3% was recorded in the original paper, but it should be revised as 56.9%. e, 84% was recorded in the original paper, but it should be revised as 30.7%. f, 45.4% was recorded in the original paper, but it should be revised as 90.3%. g, 91.1% was recorded in the original paper, but it should be revised as 46.5%. h, 41% was recorded in the original paper, but it should be revised as 89.2%. | | | | | | | | | | | | | | | | | | | | | | | | | |
